# A critical assessment of the Protoaurignacian lithic technology at Fumane Cave and its implications for the definition of the earliest Aurignacian

**DOI:** 10.1371/journal.pone.0189241

**Published:** 2017-12-07

**Authors:** Armando Falcucci, Nicholas J. Conard, Marco Peresani

**Affiliations:** 1 Department of Early Prehistory and Quaternary Ecology, University of Tübingen, Schloss Hohentübingen, Tübingen, Germany; 2 Tübingen-Senckenberg Center for Human Evolution and Paleoecology, Schloss Hohentübingen, Tübingen, Germany; 3 Università di Ferrara, Dipartimento di Studi Umanistici, Sezione di Scienze Preistoriche e Antropologiche, Corso Ercole I d'Este, Ferrara, Italy; Max Planck Institute for the Science of Human History, GERMANY

## Abstract

In the scenario of the spread of the anatomically modern humans (AMHs) into Europe, the techno-complex known as Protoaurignacian is defined by the production of blades and bladelets within a single and continuous stone knapping sequence from the same core as the result of its progressive reduction. However, the growing re-evaluation of some assemblages is revealing that bladelets are frequently obtained from independent reduction sequences, hence discouraging the direct application of the model developed in southwestern France. High-resolution regional signatures are thus needed to reconstruct a more accurate portrait of the AMH colonization dynamic. Northeastern Italy, with the key site of Fumane Cave, is one among the regions of Mediterranean Europe worthy of consideration for reconstructing this colonization process and its cultural dynamics. Within the framework of a critical discussion of the technological definition of the Protoaurignacian and its relationship with contemporaneous industries on a regional and supra-regional scale, we present the results of a detailed analysis of the lithic technology from units A2-A1 based on reduction sequence and attribute analyses. Results show that bladelets are the first goal of production and they do not originate from reduced blade cores but from a broad range of independent and simultaneous core reduction strategies. One implication is that the most commonly used technological trait that is said to define the Protoaurignacian has been over-emphasized and that the Protoaurignacian is technologically consistent across its geographical extent. Additional data based on carinated core technology imply that this techno-complex shares a common technological background with the Early Aurignacian and that no features are restricted to one of the two facies. Furthermore, the major difference between the Protoaurignacian and Early Aurignacian appears to be more typological in nature, with retouched bladelets being less common in the Early Aurignacian.

## Introduction

The Aurignacian is considered the result of the spread of anatomically modern humans (AMHs) across Europe [1–4]. To trace this migration route, the techno-complexes which are said to represent the precursors of the classic Aurignacian, like the Mediterranean Protoaurignacian and the Kozarnikian, have at times been assigned to the Early Ahmarian [5, 6]. The issue is however open to debate because of currently available chronology in the Near East [7], and the absence of a detailed comparison between techno-complexes. According to some researchers, the appearance of the Aurignacian sensu lato might represent a second wave of AMHs moving across Western Eurasia [5]. The first wave would be associated with the Bohunician in Europe, whose material culture is comparable to the Levantine Initial Upper Paleolithic [8–11]. Similar claims have been made for the Uluzzian after the assignment of two teeth to *Homo sapiens* at Cavallo cave [12]. The integrity of the Cavallo stratigraphy has, however, been questioned [13] and further evidence is needed to assess the makers of the Uluzzian industry [14, 15].

To date, the Aurignacian is the sole, undisputed techno-complex associated to AMHs [3, 16, 17]. The appearance of the Aurignacian at Willendorf II, Geißenklösterle, and Peskő dates back to about 43 ka cal BP [18–22]. Slightly later dates (c. 42 ka cal BP) exist at Isturitz [23], Mochi [24], and Arbreda [25]. The Aurignacian thus seems to overlap for few millennia with the transitional industries and late Mousterian techno-complexes [25–27]; but see Davies et al. [21].

The earliest phases are known as Protoaurignacian and Early Aurignacian (see a background history in [28, 29–35]). The Protoaurignacian was first described by Laplace [33] along the Mediterranean boundaries and in the French Pyrenees. In these regions, the Protoaurignacian is stratigraphically placed below the Early Aurignacian when both industries are documented [35–38]. According to this evidence and with the support of a series of radiocarbon dates, Banks, d'Errico and Zilhao [39] have concluded that the changes in the Early Aurignacian material culture represent the response of AMHs to the deterioration of the environment at the onset of the Heinrich event 4 (contra [40, 41]). On a supra-regional scale, however, this theory is questioned by the manifestation of the Early Aurignacian prior to HE4 in Central Europe [18–21]. Some have proposed that the two Aurignacian varieties have developed in different geographical domains and have spread across Europe along two different routes [3, 42]. The Danube represented a preferential corridor for the diffusion of Early Aurignacian industries [20], while the Mediterranean coastline was followed by makers of Protoaurignacian industries [43, 44]. These considerations raise questions about how these two apparent sister groups relate and if the assumptions that were made are consistent with the available archaeological data [45].

The Aurignacian was initially defined by the association of stone and organic tools discovered in southwestern France, with technological features subsequently investigated to isolate two distinct technical traditions [35, 46–48]. The Protoaurignacian technological signature is said to lie in the production of blades and bladelets within a single and continuous stone knapping sequence. Both products are thus obtained from the same core as the result of its progressive reduction [35, 49]. Blades are selected to manufacture end-scrapers, burins, and laterally-retouched tools. Slender blades, representing the intermediate products between blades and bladelets, are frequently left unretouched. Bladelets are the dominant intention of the lithic production and are described as large, with rectilinear profiles, and are transformed into Dufour sub-type Dufour [50]. The Early Aurignacian is instead characterized by a clear distinction between laminar and lamellar productions as result of a stronger anticipation and planning of different needs [51, 52]. Blades are obtained from unidirectional prismatic cores, while curved bladelets are produced from carinated cores, frequently called “carinated end-scrapers” (see a research history in [53]). The latter are said to be scarcely found, or even absent, in Protoaurignacian assemblages [36]. Blades are robust, have frequently faceted platforms, and are transformed into laterally-retouched tools, strangled blades, and thick end-scrapers. These common tools are often modified by the so-called Aurignacian retouch [31], which is scalar and invasive due to several re-sharpening stages that occur during repeated use and transport over long distances [54]. Bladelets are instead produced on-site, as needed, and only few were transformed into small sub-type Dufour [55].

Aside from stone tools, historically, the most important type-fossil associated with the Early Aurignacian is the split-based bone point [31, 48]. Recently, the exclusive association of split-based bone points with Early Aurignacian assemblages has been questioned and its presence in an archaeological horizon does not in and of itself clarify the cultural attribution [56, 57]. At Geißenklösterle, for instance, split-based bone points appear only in the upper Early Aurignacian horizon [20, 51], while at Trou de la Mère Clochette [58] and Arbreda [59] split-based bone points were found in association with Protoaurignacian lithic implements.

Additionally, the Early Aurignacian has produced three-dimensionally formed personal ornaments, figurative representations, occasional finds of mythical imagery, and musical instruments, whereas the Protoaurignacian typically has a more limited range of symbolic artifacts, made especially on marine shells and animal teeth [60–63].

The growing number of multi-disciplinary analyses and the re-evaluation of some assemblages are highlighting a greater technological variability that is casting serious doubts on the direct application of the model developed in southwestern France. Lithic assemblages with mixed features have been described in the Basque Country, Romania, and Crimea [23, 56, 64, 65]. Also, technological analyses carried out at some Protoaurignacian sites have revealed that bladelets are frequently obtained from independent reduction sequences [46, 56, 66]. As noticed by Bon [35], a further step in the research history is needed in order to build up high-resolution Aurignacian regional signatures and to reconstruct a more accurate portrait of AMHs colonization dynamics.

Here, we present a detailed analysis of the lithic technology of the Protoaurignacian from units A2-A1 of Fumane Cave in northeastern Italy. Fumane has always been considered a key site for understanding the Middle-to-Upper Paleolithic transition and the complex processes that led to the demise and final extinction of Neandertal populations and the spread of AMHs across Europe. The systematic and modern excavations conducted for decades, the presence of a high resolution stratigraphic sequence that includes the Mousterian, the Uluzzian, and the Protoaurignacian, and the discovery of modern human remains associated with the Protoaurignacian [17], allow us to critically discuss the technological definition of this techno-complex and its relationship with contemporaneous industries on a regional and supra-regional scale. Previous studies on the lithic assemblage [43, 67] have the merits of having described the variability of bladelet production, even if additional quantitative research was needed to discuss in detail the procedures and the objectives of the stone knapping. Specifically, we present the results of an extensive investigation on the Protoaurignacian lithic technology by using two combined approaches: reduction sequence and attribute analyses. The information gained during the analytical process will be then compared with the existing literature, in order to address the following research questions:

1. What are the main goals of the Protoaurignacian lithic technology at Fumane Cave and how are they met?
2. Is the continuous reduction sequence theory [48] a viable proxy to define the Protoaurignacian on a technological ground?
3. What are the shared features of Protoaurignacian lithic technology across its geographical extent?
4. How does the Protoaurignacian relate to the Early Aurignacian, and how do the archaeological data fit with the reconstruction proposed by Banks, d'Errico and Zilhao [39]?

## Fumane Cave, the Middle-to-Upper Paleolithic transition, and the Aurignacian

**Fig 1:**
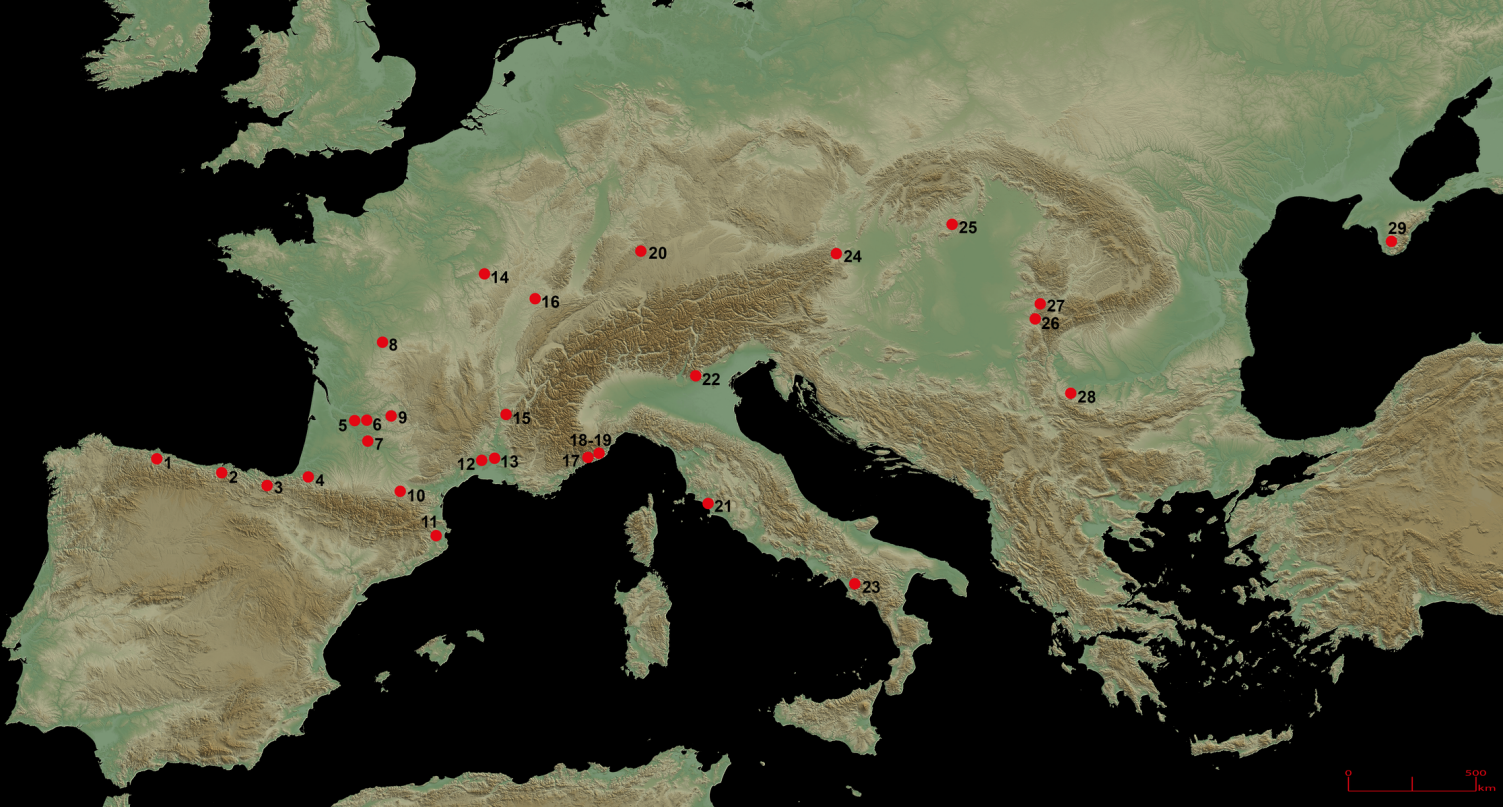
Map showing the localization of Fumane Cave and other Aurignacian sites cited throughout the paper. 1 = La Viña (Spain), 2 = Morin (Spain), 3 = Labeko Koba (Spain), 4 = Isturitz (France), 5 = Champ-Parel (France), 6 = Barbas III (France), 7 = Hui (France), 8 = Les Cottés (France), 9 = Piage (France); 10 = Tuto-de-Camalhot (France), 11 = Arbreda (Spain), 12 = Esquicho-Grapaou (France), 13 = Louza (France), 14 = Arcy (France), 15 = Mandrin (France), 16 = Trou de la Mère Clochette (France), 17 = Observatoire (France), 18 = Mochi (Italy), 19 = Bombrini (Italy), 20 = Geißenklösterle (Germany), 21 = La Fabbrica (Italy), 22 = Fumane (Italy), 23 = Castelcivita (Italy), 24 = Willendorf II (Austria), 25 = Peskő (Hungary), 26 = Tincova (Romania), 27 = Româneşti (Romania), 28 = Kozarnika (Bulgaria), 29 = Siuren I (Crimea). Map downloaded from the NASA Earth Observatory (http://earthobservatory.nasa.gov/) and processed by K. Di Modica (Scladina Cave Archaeological Center).

Fumane Cave, excavated since 1988, lies at the foot of the Monti Lessini Plateau (Venetian Prealps; Fig 1). Details about the cave’s structure, Late Pleistocene stratigraphic sequence, and paleoclimatic significance, as well as its paleontological and cultural content, are available in numerous publications [15, 17, 67–72]. A main cave and two associated tunnels preserve a finely-layered sedimentary succession spanning the late Middle Paleolithic and the Early Upper Paleolithic, with features and dense scatters of remains in units A11, A10, A9, and A6–A5 (Mousterian [71, 73]), A4 and A3 (Uluzzian [15, 74]), A2 and A1 (Protoaurignacian [43, 67, 75]), D6 and D3 (Aurignacian lato sensu [68]). Currently, layers A9 to A1 have been extensively excavated at the entrance of the cave and partly excavated in the cave mouth.

In layers A4 and A3, the Uluzzian occupations date to later than 43.6–43.0 ky cal BP [69]. The transition from the final Mousterian took place in a relatively short time, as the beginning of the Uluzzian is chronologically indistinguishable from the final Mousterian [27]. The Uluzzian lithic technology is primarily oriented towards flake production. Technological innovations are rooted in a clear Mousterian cultural context [15]. In layer A4, flakes are obtained from centripetal cores, following Levallois concepts. Scrapers of varied morphologies are the prevailing tool type. Layer A3 marks the definitive separation of the Uluzzian from the Mousterian. In this layer, flakes are produced through several methods and bladelet production slightly increases. The main tool types are scrapers, splintered pieces, and backed flakes.

Unit A2 dates the appearance of the Protoaurignacian to 41.2–40.4 ky cal BP [69]. Its boundary with layer A3 and with the overlying layer D3 is clear and is marked by a dispersion of ocher over a large extent of the area [75, 76] and by a considerable change in the content of anthropogenic material [77]. In the cave entrance, unit A2 is covered by unit A1, a thin anthropic level with horizontal bedding which makes it indistinguishable from A2 in the cave mouth. A2 thus extends throughout the whole cave extent.

Post-depositional processes, due to frost activity, affected layers A3 and A2 in the easternmost part of the cave entrance and produced infiltrations of Protoaurignacian materials (lithics, bones, and shells) into A3 [15]. Stratigraphic deformations have been reported in the inner eastern side of the cave mouth, where layer A2 was tilted and compressed towards the cave wall, forming a pronounced fold. Despite this deformation, during the excavation layer A2 appeared like a clearly discernible sedimentary body preserved with variable thickness from a few to 10 centimeters, due to its dark-brownish color, its texture and its high charcoal, bone and stone implement density, as well as the occurrence of features (i.e. hearths, post-holes, toss-zones) mostly located at the cave entrance [78, 79]. Some of these hearths were located within shallow basins excavated at the expenses of the Uluzzian and final Mousterian layers below, thus producing possible dispersion of few flaked stones in the A2-A1 Protoaurignacian assemblage.

The consistency of A2-A1 assemblages is also secured by the lack of any evidence supporting massive percolation of stone implements from the above D6-D3 stratigraphic complex and related layers at the cave entrance. Clear boundaries between Aurignacian contexts, as well as the lack of deformations, point for excluding a mixing between different Aurignacian occupations. The youngest Aurignacian phase is from the stratigraphic complex D6-D3, which includes several layers embedded in coarse-sandy sediments. Layers D3a and D3b are the most extended, while D6 is a loose stony layer limited to the eastern zone of the cave. The traces of human presence are less dense than in A2-A1, however, hearths and other surface features have been exposed.

Ornamental objects represent a regular cultural component of the Aurignacian layers. They consist of grooved red deer incisors and several hundreds of perforated shell beads belonging to sixty different taxa, most of them marine [68, 80]. The bone and antler industry is composed of a variety of tools [43, 68]. Split-based bone points are not found in units A2-A1; they are only found in units D6 and D3, except one implement found at the interface between D3 and A1 [43]. The same is true of the five rock fragments painted with red ocher [68, 77]. The lithic implements of units D6-D3 do not seem to differ significantly from A2-A1 [67, 69, 81]. New, careful, investigations are being performed by one of us (AF) to test this first hypothesis.

Faunal remains shed lights on the Aurignacian ecological context. They show an association between forest fauna and cold and open habitat species typical of the alpine grassland steppe above the tree line [82]. This context reflects a clear climatic cooling with relative decreases in woodland formations, as also indicated by the micromammal associations [70].

## Materials and methods

Units A2 and A1 do not show significant differences on typo-technological or chronological grounds [69], and were undistinguishable in the cave mouth during the excavations. For these reasons and for the purpose of this study, it was considered more accurate to incorporate both layers into a single analytical unit. The archaeological material was either directly excavated using a 33×33 cm grid or recovered from wet sieving. All artifacts, independently from their size, are available for detailed investigations; except for a small set of cores (n = 5) and tools (n = 17) that are on display in permanent exhibitions at the Museo Paleontologico e Preistorico di Sant’Anna d’Alfaedo. In order to conduct an extensive technological analysis of the Protoaurignacian lithics, all artifacts greater than 1.5 cm in maximal dimension were counted (A2 = 22,212; A1 = 4,153 items) and divided according to several technological classes and the sub-square of provenience. The minimal number of flaked products (MNFP), which was calculated by taking into account only blanks with preserved butts, permitted a better estimation of the amount of lithics. This step was judged necessary because no previous quantitative analysis of the lithic assemblage had been undertaken. The data gained during this first phase was used to evaluate the frequency of technological categories and the degree of cortex extension on artifacts. The sampling procedure is based on the dispersion of lithic materials in the squares and an evaluation of the stratigraphic context, as described in the excavation notebooks. Seven square meters were selected (S1 Fig). They are located in different sectors of the cave and are close to the main combustion features. Two adjacent square meters were analyzed in those sectors with the highest concentration of lithics. Early on in the study it became clear that A2-A1 is a blade-bladelet dominated industry. For this reason, all blades and bladelets greater than 1.5 cm in maximal dimension, regardless of the degree of fragmentation, were analyzed, while only flakes with preserved butts greater than 2.0 cm in maximal dimension were fully analyzed. Furthermore, the extent of the cave was sampled in order to isolate and include in the database all cores, tools and tool fragments, all complete and almost complete blades and bladelets, and all by-products deemed to have had a significant role in the reduction process. Only the innermost part of the cave, affected by a stratigraphic deformation (see above), was excluded from the analysis. This strategy was considered effective to avoid potential biases in the reconstruction of the blank production system. Therefore, we analyzed a total of 7,866 artifacts.

The Protoaurignacian industries have been made on flint of different carbonatic formations, which, in the western Monti Lessini, range from the Upper Jurassic to Middle Eocene. They were easily collected within 5–15 km from the site. The most widespread types, distinguished on the base of macroscopic features, are from the Maiolica, the Scaglia Rossa, the Scaglia variegata, and the Ooliti di San Virgilio formations. Flint also abounds in loose coarse stream or fluvial gravels, slope-waste deposits, and soils in the immediate surroundings of the cave [83]. Jurassic and Tertiary calcarenites, frequently found in large-sized and homogeneous nodules, were almost exclusively used to produce blades [43].

The lithic analysis approach combines two complementary methods: reduction sequence analysis [84–88] and attribute analysis [10, 89, 90]. The first permits identification of the methods of core reduction and the stages of knapping, and use and discard of stone artifacts enchained in a temporal trajectory. The second is particularly valuable because it provides quantitative data on the numerous discrete and metric features that can be recorded on individual artifacts. The attributes recorded in the database are based on recent studies and have been shown to be valuable for understanding laminar technologies at the onset of the Upper Paleolithic (e.g. [8, 91]).

Additionally, diacritic analyses [92, 93] were performed to reconstruct the chronology, the direction of removals, the stages of production on discarded cores, and short sequences of removals on blanks. By doing this, the detailed procedures of core reduction were identified [94]. Diacritic investigations have been particularly helpful to contextualize the operations and technical expedients performed to maintain the core structure and to isolate recurrent patterns among the studied assemblage.

Non-extensive refitting analyses were also conducted throughout the study. They have proven to be particularly valuable to test hypotheses formulated during the analytical process.

Supplementary and specific databases were designed to record additional features on particularly informative blank types such as core tablets and technical blanks, and also to discriminate the knapping technique (based on [95, 96]).

The unified taxonomy by Conard et al. [97] was used to give a general overview of core categories. Platform cores have been further divided into several reduction strategies according to criteria such as: orientation of the flaking surface, knapping progression, and number of platforms and faces exploited.

In order to assess the curvature of blanks, dorsal scars, and shape only complete and almost complete specimens have been taken into account. This is beneficial in that it avoids biases due to the high degree of fragmentation of the assemblage. Profile curvature was quantified using the categories defined by Bon [35]. Retouched tools were excluded from the analysis of morphology and distal ends due to the modification of the shape via retouching. The metric boundary between blades and bladelets was placed at 12.0 mm [98], in agreement with most of the studies conducted on Aurignacian assemblages and according to our case study. At Fumane, the inverse and alternate retouch, common among retouched bladelets, is indeed rarely applied on laminar tools wider than 12.0 mm (n = 16; 3.9%).

The maximum dimensions of each artifact were recorded using a digital caliper and metric differences were assessed in IBM SPSS Statistics 24. Given that our sample was not normally distributed according to Shapiro–Wilk and Kolmogorov–Smirnov tests, we have performed non-parametric tests (Mann–Whitney and Kruskall–Wallis). Given that multiple tests were conducted, the Holm–Bonferroni sequential correction test was utilized for the purpose of reducing the probability of performing a type 1 error [99].

## Results

### Quantitative analysis of the knapped assemblage

The quantitative analysis of the knapped assemblage (Table 1) shows that blanks dominate, followed by tools, angular debris, and, finally, cores. The paucity of cores is not surprising and may be explained as the result of a high on-site reduction, but also as an off-site transport of non-exhausted cores. Seven raw materials were discarded prior blank production, after at least one removal that aimed to evaluate the quality of the selected piece. Tested raw materials have maximum linear dimensions (MLD [89]) that range from 63.7 to 111.9 mm (mean: 82.5 mm), polygonal morphologies, and are almost completely cortical.

**Table 1:** Quantification of the knapped assemblage (> 1.5 cm).

Category	Number	Percentage
**Blank**	21373	81
**Tool**	3177	12
**Core**	155	0.6
**Angular debris**	1674	6.3
**Tested nodule**	7	-
**Total**	26386	100

Table 2 summarizes the frequency of the main blank types and gives a detailed technological overview among each class. The frequency of by-products related to maintenance operations may be underestimated due to the degree of fragmentation. Only specimens with a combination of technologically relevant attributes have been typed under specific sub-types. Laminar products dominate the blank assemblage. Taken together, blades and bladelets amount to 63.3% (MNFP = 62.2%). Flakes are relatively abundant, even if this category is mainly composed of by-products of blade and bladelet reduction strategies (see below). The degree of breakage is high (90.1%), while MNFP amounts to 48.8% of the entire blank assemblage. Cortical surfaces are well-represented among flake (22.2%) and blade (20.2%) categories, while among bladelets, they are rare (5.3%). This evidence suggests that raw material decortication and core initialization resulted mostly in the production of flakes and blades of variable sizes. Among the studied sample, the decortication phase is represented by objects with more than 66% cortex coverage (n = 198). Most of the pieces are flakes (n = 118), followed by blades (n = 66), and rarely bladelets (n = 14). There is no significant difference between size and cortex when the length of complete blanks is compared across specimens with different grades of cortex coverage (S1 Table; Kruskall–Wallis, H = 1,163; p = 0.7).

**Table 2:** Distribution of blank types (> 1.5 cm) according to the whole assemblage and the minimal number of flaked products (MNFP).

Blank type	Number	MNFP
**Flake**	**8921 (36.3%)**	**4486 (37.4%)**
Flake	6671 (74.8%)	3321 (74.0%)
Semi-cortical flake	1347 (15.1%)	631 (14.1%)
Fully cortical flake	499 (5.6%)	178 (4.0%)
Debordant flake	69 (0.8%)	61 (1.4%)
Crested flake	8 (0.1%)	7 (0.2%)
Two-sided crested flake	2 (-)	2 (-)
Crested secondary flake	1 (-)	1 (-)
Neo-crested flake	6 (0.1%)	4 (0.1%)
Technical flake	149 (1.7%)	120 (2.7%)
Lateral comma-like flake	5 (0.1%)	4 (0.1%)
Core tablet	164 (1.8%)	157 (3.5%)
**Blade**	**5875 (23.9%)**	**2941 (24.5%)**
Blade	4460 (75.9%)	2214 (75.3%)
Semi-cortical blade	913 (15.5%)	410 (13.9%)
Fully cortical blade	99 (1.7%)	43 (1.5%)
Naturally backed blade	68 (1.2%)	49 (1.7%)
Crested blade	35 (0.6%)	16 (0.5%)
Two-sided crested blade	13 (0.2%)	8 (0.3%)
Crested secondary blade	36 (0.6%)	22 (0.7%)
Neo-crested blade	51 (0.9%)	32 (1.1%)
Technical blade	117 (2.0%)	86 (2.9%)
Lateral comma-like blade	83 (1.4%)	61 (2.1%)
**Bladelet**	**9664 (39.4%)**	**4513 (37.7%)**
Bladelet	9009 (93.2%)	4237 (93.9%)
Semi-cortical bladelet	509 (5.3%)	185 (4.1%)
Fully cortical bladelet	11 (0.1%)	3 (0.1%)
Crested bladelet	36 (0.4%)	15 (0.3%)
Two-sided crested bladelet	2 (-)	2 (-)
Crested secondary bladelet	22 (0.2%)	14 (0.3%)
Neo-crested bladelet	17 (0.2%)	8 (0.2%)
Technical bladelet	32 (0.3%)	26 (0.6%)
Lateral comma-like bladelet	26 (0.3%)	23 (0.5%)
**Burin Spall**	**80 (0.3%)**	**49 (0.4%)**
**Undetermined**	1**0 (-)**	**-**
**Total**	**24550 (100%)**	**11989 (100%)**

The count includes blank types of tools. Percentages are given in brackets.

### Core reduction

Three core reduction methods were identified in layers A2-A1: platform, multidirectional, and parallel. Platform cores represent the most abundant category, with multidirectional and parallel reduction strategies playing a secondary role (Table 3). Core fragments belong mostly to platform cores, even if most of them cannot be further sub-grouped. Knappers employed multidirectional and parallel methods to produce flakes of varied morphologies and used the platform method to obtain blades and bladelets. Some evidence suggests that platform cores were sometimes recycled to produce flakes from two or more core faces, obliterating the previous removal scars. This is the case of a discarded blade core, and of a blade core fragment. In the following paragraphs the three core reduction strategies are described.

**Table 3:** Distribution of core categories.

Core category	Number
Initial platform core	26 (16.8%)
Platform	89 (57.4%)
*Narrow-sided*	*23 (25*.*8%)*
*Semi-circumferential*	*20 (22*.*5%)*
*Wide-faced flat*	*13 (14*.*6%)*
*Transverse carinated*	*10 (11*.*2%)*
*Multi-platform*	*23 (25*.*8%)*
Parallel	5 (3.2%)
Multidirectional	9 (5.8%)
Core fragment	26 (16.8%)
**Total**	155 (100%)

Platform cores are further divided according to the five reduction strategies identified. Percentages are given in brackets.

#### Multidirectional cores

In the case of Fumane, this group includes cores that have removals from two or more faces without well-developed striking platforms. They have polyhedral morphologies, and display irregular negatives of removals. All of them have produced flakes by rotating the cores according to the exploitable morphology achieved after the former removals. One of these cores exploited a fragment of a blade core, identified thanks to the preservation of a portion of the striking platform and a few related unidirectional scars which were almost completely covered by the flake negatives. Multidirectional cores have produced from three to six flakes prior to discard. The negatives of bulbs suggest that flakes were detached by using direct internal percussion, without any particular kind of preparation prior detachment. To conclude, this core reduction strategy seems to be rather opportunistic and marginal.

#### Parallel cores

**Fig 2:**
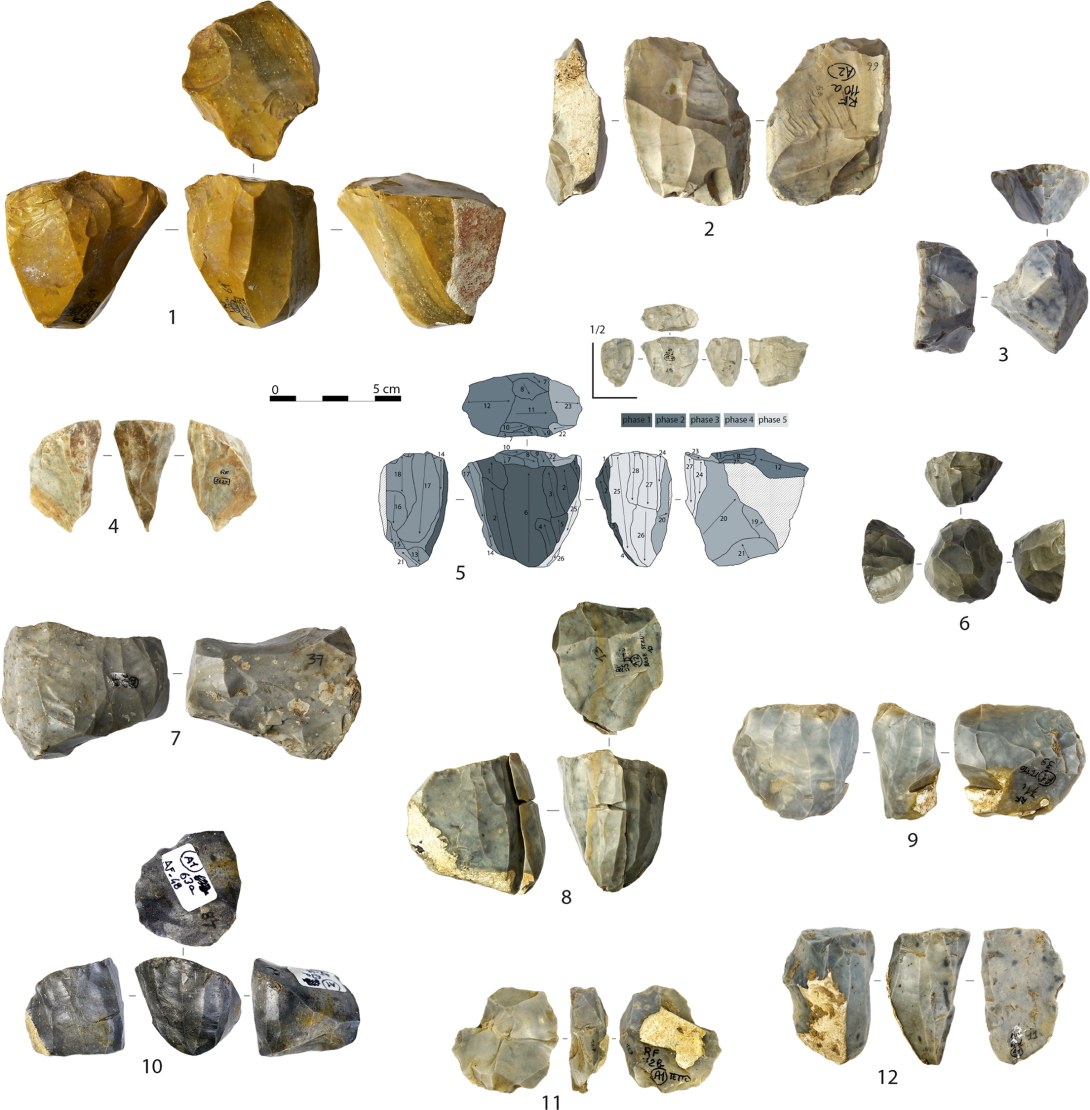
Cores. Semi-circumferential blade core (1), wide-faced flat blade core with scars of a technical orthogonal flake on the proximal side (2), transverse carinated cores (3, 6), narrow-sided cores (4, 12), multi-platform core, and its schematic drawing (arrows indicate direction of the removals and numbers indicate the order of the removals), exploited for blade (phase 1) and bladelet productions (phases 3 and 5) (5), wide-faced flat core with evidence of a simultaneous production of small blades and big bladelets (7), semi-circumferential bladelet core with a refitted plunging blade (8), multi-platform bladelet core exploited on the narrow face and successively on the wide face in two distinct phases (9), semi-circumferential bladelet cores (10), and parallel flake core (11) (photo and drawing: A. Falcucci).

Parallel cores are characterized by a removal surface with centripetal negatives that originated from the intersection with the underside (Fig 2: 11). This underside presents short platform preparation scars all along its periphery, while its central area is always cortical. In two cases, the striking platform is weakly trimmed. The flaking angle is around 70° to 80° and the pronounced bulbar negatives relate with the application of direct internal percussion. The final size of the cores suggests a high degree of reduction (mean MLD = 39.2 mm). Last removal scars suggest that, through this method, knappers obtained polygonal flakes, some of them characterized by hinged distal terminations. This reduction method must be treated with caution, due to its strong resemblance to the centripetal flake method of the Uluzzian layers A4 and especially A3 [15]. On the other hand, the spatial distribution analysis shows that parallel cores were found in different sectors of the cave, making the attribution to A2–A1 at least plausible.

#### Platform cores

Platform methods were used to manufacture almost exclusively blades and bladelets. Cores have been discarded at different stages of reduction. Exhausted platform cores can be classified as blade cores (n = 6), bladelet cores (n = 76), blade-bladelet cores (n = 5), and blade-flake cores (n = 1) according to the organization of the last visible scars. One core is undetermined. Bladelet cores may display laminar scars wider than 12.0 mm related to maintenance operations. For this reason, they have not been typed as blade-bladelet cores. The latter are characterized by a clear alternation of blade and bladelet removals, or by an independent bladelet production performed on a re-oriented blade core. Finally, initial platform cores were identified. Under this category, all objects displaying only few removal scars have been included. They reflect the initial stages of knapping in which much of the original piece is still unmodified. Initial platform cores represent an important source of information because they allow appreciation of the preliminary flaking and configuration of the selected blanks before their overall morphology is modified and the volume is reduced. The lengths of the flaking surfaces suggest that most of them were intended to be bladelet cores. Only five specimens, ranging from 55.6 to 116.1 mm (mean: 76.5 mm), may have served as blade cores. On the other hand, initial bladelet cores frequently display shaping negatives that belong both to blades and flakes. Five reduction strategies were identified among platform cores [94]. Their main features can be summarized as follows:

1. Narrow-sided core This category consists of cores exploited on the narrow face along the longitudinal axis to produce exclusively bladelets (Fig 2: 4,12). They are made from flakes or flat raw material nodules selected according to their thickness and are frequently characterized by posterior crests or dorsal thinning.
2. Semi-circumferential core This category corresponds to cores that have been exploited along the longitudinal axis around at least two available sides in continuity, by turning the core during the reduction process (Fig 2: 1,8,10). Semi-circumferential cores can have a rectangular or triangular removal surface. They have produced bladelets (n = 15), blades (n = 4), and blades and bladelets simultaneously (n = 1).
3. Wide-faced flat core The third category is composed of cores exploited in one of the broader faces of the blank, along the longitudinal axis (Fig 2: 2,7). They have been discarded in an advanced stage of reduction, given that at least one of the flanks is missing, linking the flaking surface directly to the back of the core. Last removals at discard correspond to blades (n = 2), to a simultaneous blade and bladelet production (n = 1), and especially to bladelets (n = 9). One core is undeterminable due to a technical flake that obliterated the previous removal scars.
4. Transverse carinated core This category groups cores that have been oriented on the transversal axis to exploit the thickness of the available blank (Fig 2: 3,6). They have technological attributes comparable to well-known descriptions (see in [35, 46]) and are distinct from the rest of the categories because the frontal regression of the knapping penetrates orthogonally along the longitudinal axis of the blank. Core thickness corresponds to the length of the former categories. Transverse carinated cores are made almost exclusively from flakes and bladelets are the goal of the production.
5. Multi-platform core This core category is the most variable, being composed of cores exploited on one or more faces, starting from two or more platforms during independent reduction stages (Fig 2: 5,9). Last visible scars display bladelet removals most often (n = 19), simultaneous blade and bladelet removals followed by a disjointed bladelet production (n = 1), bladelets with a previous and disjointed blade production (n = 2), and blades followed by flakes (n = 1).

**Fig 3:**
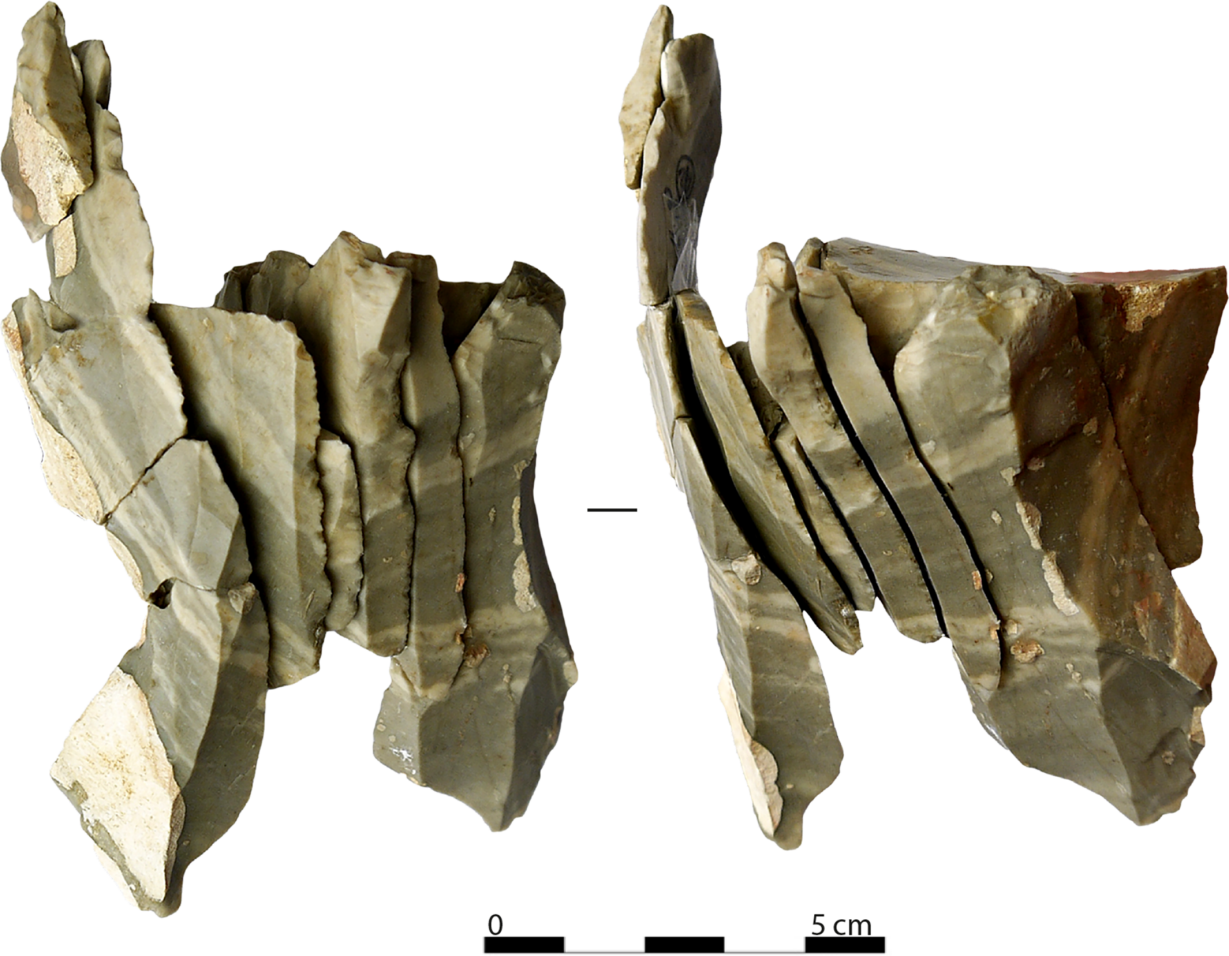
Refitted semi-circumferential blade core (photo: A. Falcucci).

Globally, platform cores represent a relatively homogenous category, where all the identified sub-categories share a certain degree of technological overlap (see core schematic drawings and diacritic analyses in S2 Fig). Two core types, narrow-sided and transverse carinated cores, have been used exclusively to produce bladelets. Blade cores are found in the other categories. Their length at discard does not exceed 66.4 mm. A refitted blade core (Fig 3) provides an example of reduction intensity. Its length at discard is 36.4 mm, while its refitted length is 105.3 mm. Among blade cores, a sub-parallel reduction pattern is exclusive, while a convergent reduction pattern is well attested among to bladelet cores. Overall, the progression of knapping is parallel to the axis of core symmetry and is always unidirectional. Opposed platforms were sometimes used to maintain the core distal convexity (n = 11).

**Fig 4:**
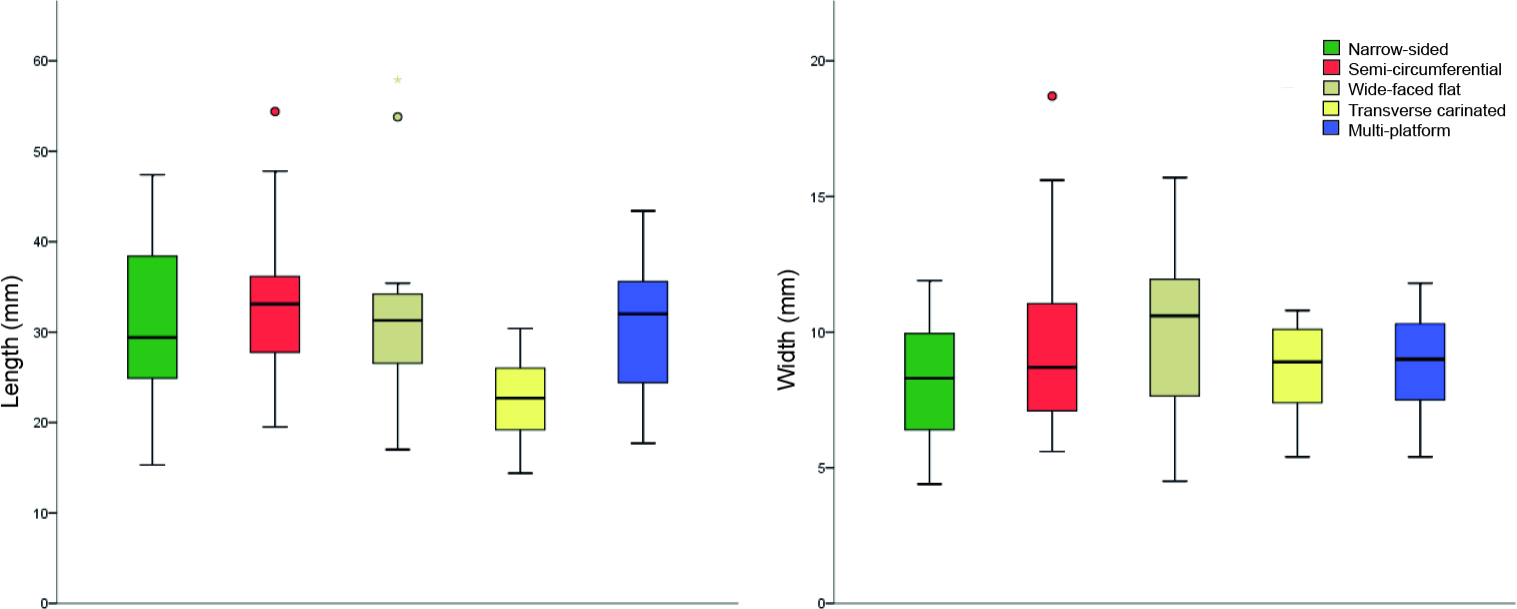
Box-plots of length (left) and width (right) values (in millimeters) of the last complete negatives measured on platform cores divided per reduction strategy. For colors see the legend.

The last complete removals across platform core sub-categories are compared in Fig 4. The dimensions of the last complete negatives are similar for all core sub-categories, with only transverse carinated cores displaying shorter removals and narrow-sided cores targeting slender bladelets.

### Overall blank analysis

#### Blades and bladelets

**Fig 5:**
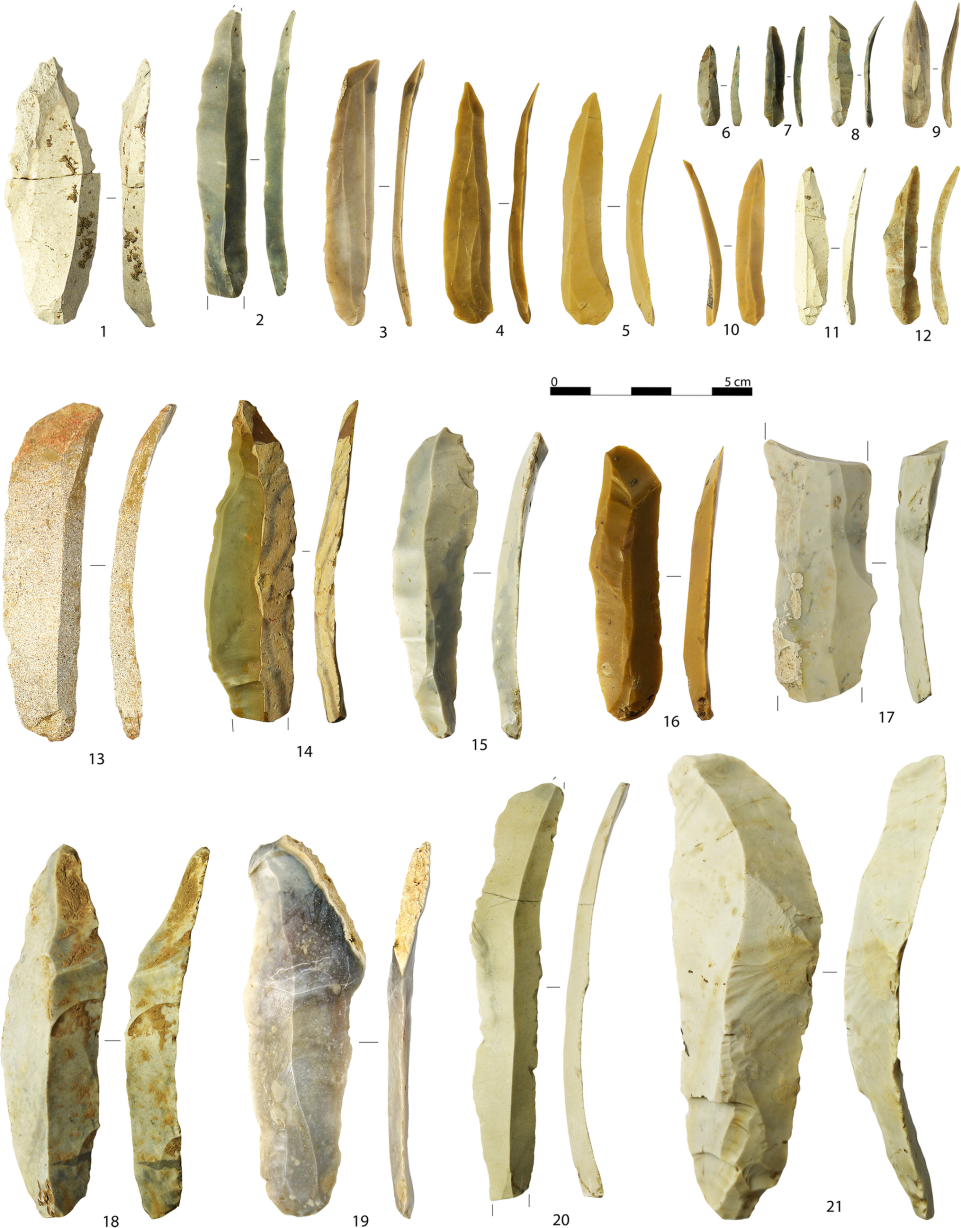
A sample of blades (1, 13–21) and bladelets (2–12) of different sizes with unidirectional scar patterns. Artifacts are oriented with the butt at the bottom of the photo (photo: A. Falcucci).

Morphological and technological attributes of blades and bladelets (Fig 5) are listed in Table 4.

**Table 4:** Morphological and technological attributes of blades, bladelets, and flakes.

Morphological and technological attributes	Blade	Bladelet	Flake
Profile			
Straight	111 (20.6%)	185 (26%)	208 (40.1%)
Slightly curved	107 (19.9%)	195 (27.4%)	119 (22.9%)
Curved	138 (25.6%)	178 (25%)	108 (20.8%)
Intense curvature	69 (12.8%)	39 (5.5%)	48 (9.2%)
Inverse curvature	-	4 (0.6%)	8 (1.5%)
Twisted	114 (21.5%)	110 (15.5%)	28 (5.4%)
Orientation			
Axial	492 (82.1%)	598 (82.8%)	417 (91.6%)
Off-axis	99 (16.5%)	114 (15.8%)	36 (7.9%)
Undetermined	8 (1.3%)	10 (1.4%)	2 (0.4%)
Cross-section			
Triangular	523 (26.3%)	2030 (47.0%)	175 (13.7%)
Trapezoidal	819 (41.2%)	1756 (40.6%)	294 (23.1%)
Polyhedral	317 (16.0%)	180 (4.2%)	95 (7.5%)
Lateral steeped	254 (12.8%)	261 (6.0%)	230 (18.0%)
Rectangular	13 (0.7%)	12 (0.3%)	204 (16.0%)
Flat	57 (2.9%)	80 (1.9%)	272 (21.3%)
Undetermined	4 (0.2%)	3 (0.1%)	5 (0.4%)
Cross-section symmetry			
Symmetrical	1561 (78.6%)	3930 (90.9%)	928 (72.8%)
Asymmetrical	426 (21.4%)	392 (9.1%)	347 (27.2%)
Dorsal scar pattern			
Unidirectional sub-parallel	292 (54.2%)	340 (47.8%)	222 (42.8%)
Unidirectional convergent	129 (23.9%)	302 (42.5%)	59 (11.4%)
Unidirectional transverse	59 (10.9%)	49 (6.9%)	63 (12.1%)
Bidirectional	32 (5.9%)	14 (2.0%)	31 (6.0%)
Crossed	8 (1.5%)	3 (0.4%)	62 (11.9%)
Other	19 (3.5%)	3 (0.4%)	82 (15.8%)
Outline morphology			
Sub-parallel	229 (52.2%)	249 (44.5%)	143 (34.5%)
Convergent	60 (13.7%)	196 (35.1%)	31 (7.5%)
Irregular	150 (34.2%)	114 (20.4%)	241 (58.1%)
Distal end—dorsal view			
Straight	142 (23.7%)	81 (11.2%)	151 (33.2%)
Pointed	104 (17.4%)	334 (46.3%)	35 (7.7%)
Convex-concav	279 (46.6%)	267 (37%)	160 (35.2%)
Irregular	62 (10.4%)	29 (4.0%)	99 (21.8%)
Undetermined	12 (2.0%)	11 (1.5%)	10 (2.2%)
Distal end—profile view			
Feathered	367 (61.3%)	639 (88.5%)	237 (66.8%)
Stepped	95 (15.9%)	43 (6.0%)	114 (32.1%)
Plunging	108 (18.0%)	22 (3.0%)	65 (18.3%)
Hinged	17 (2.8%)	7 (1.0%)	29 (8.2%)
Undetermined	12 (2.0%)	11 (1.5%)	10 (2.8%)

Note that profile curvature, dorsal scar pattern, and outline morphology attributes take into account only complete and almost complete specimens. Retouched tools are excluded from the analysis of the outline morphology and distal end on dorsal and profile views. Percentages are given in brackets.

Curved profiles, of different intensity grades, clearly dominate the blade and bladelet samples. Straight profiles are more common among bladelets, while the frequency of intense curved blanks is higher among blades. Twisted specimens are common, especially across blades. Twisting is, in most cases, slightly pronounced for both blades (67.5%) and bladelets (67.3%), and is usually associated with an off-axis orientation of the blank. Twisted specimens are likely to have been produced from the periphery of the core flaking surface, especially for maintenance operations.

Cross-sections are mainly trapezoidal and triangular in shape. In the bladelet category, however, triangular cross-sections are dominant, indicating that a single ridge was frequently used during knapping. Polyhedral and lateral steeped cross-sections are more common among blades and, in most cases, characterize technical and naturally backed blades. Symmetrical cross-sections dominate both groups, but asymmetrical specimens are more frequent among blades.

Dorsal scar pattern is strictly unidirectional, with few occurrences of bidirectional scars. Blades and bladelets with bidirectional scar patterns indicate the use of opposed platforms to maintain the distal side of the core. In other cases, they characterize the first removals from an opposed platform during a new reduction stage, as shown by multi-platform cores. The major difference between categories is the relevance of the unidirectional convergent scar pattern across bladelets. Bladelets with convergent scars have almost the same importance of specimens with sub-parallel scars. The presence of a transverse scar pattern testifies also to slight changes in the direction of blade and bladelet removals on the flaking surface.

Bladelets with a convergent outline morphology starting from the mesio-distal part are numerous. Furthermore, bladelets with pointed distal ends are more common than blades with pointed distal ends. In profile view, the frequency of plunging and stepped distal ends is very low among bladelets, while together they amount to 33.9% of the blades. Even if some of them are linked to striking accidents, this high frequency may be related to maintenance operations carried out from the main striking platform with the aim to remove part of the core base.

A summary of metric attributes of blade and bladelet blanks is given in Table 5.

**Table 5:** Summary of metric attributes of blades, bladelets, and blades and bladelets considered as a whole.

	Number	Range	Mean	SE	SD	25 prcntl	Median	75 prcntl
**Blade**								
Length	420	24.2 to 102.5	49.61	0.65	13.32	39.85	47.5	58.00
Width	1578	12.1 to 35.8	16.53	0.10	4.00	13.6	15.4	18.3
Thickness	1578	1.1 to 21.0	4.47	0.05	2.22	2.9	4.0	5.4
**Bladelet**								
Length	553	10.8 to 66.7	27.58	0.38	9.11	21.0	26.0	33.25
Width	1808	2.6 to 12.0	8.81	0.04	1.96	7.4	9.0	10.5
Thickness	1808	0.5 to 8.8	2.37	0.02	1.09	1.6	2.2	2.8
**Blade and bladelet**								
Length	973	10.8 to 102.5	37.1	0.049	15.58	24.85	35.0	46.5
Width	3386	2.6 to 35.8	12.41	0.08	4.93	8.8	11.5	15.0
Thickness	3386	0.5 to 21.0	3.35	0.03	2.01	2.0	2.8	4.1

SE: standard error; SD: standard deviation. Tools are excluded from the analysis.

**Fig 6:**
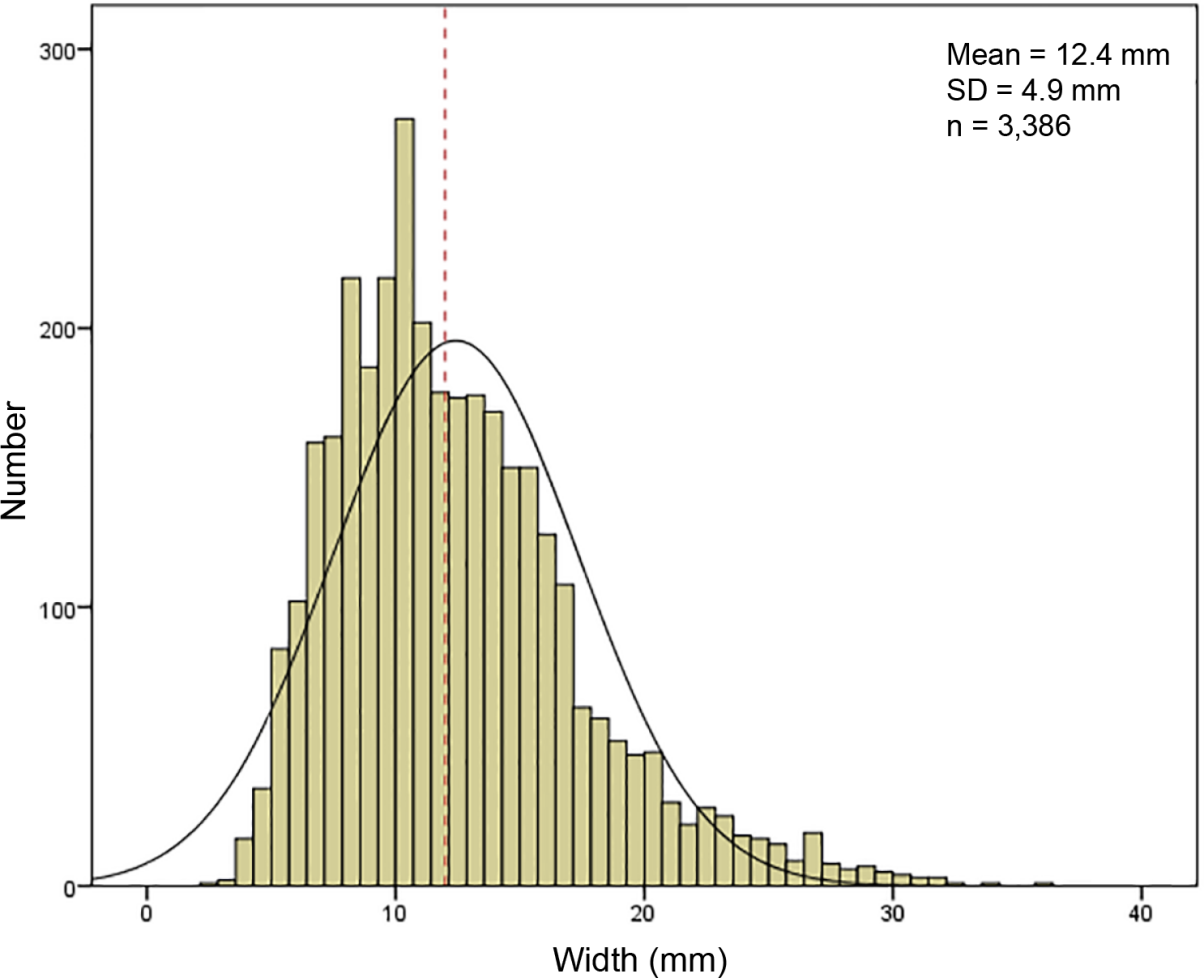
Distribution of blade and bladelet widths (in millimeters) considered as a whole. The red dashed line represents the arbitrary metric limit (12.0 mm) between blades and bladelets.

**Fig 7:**
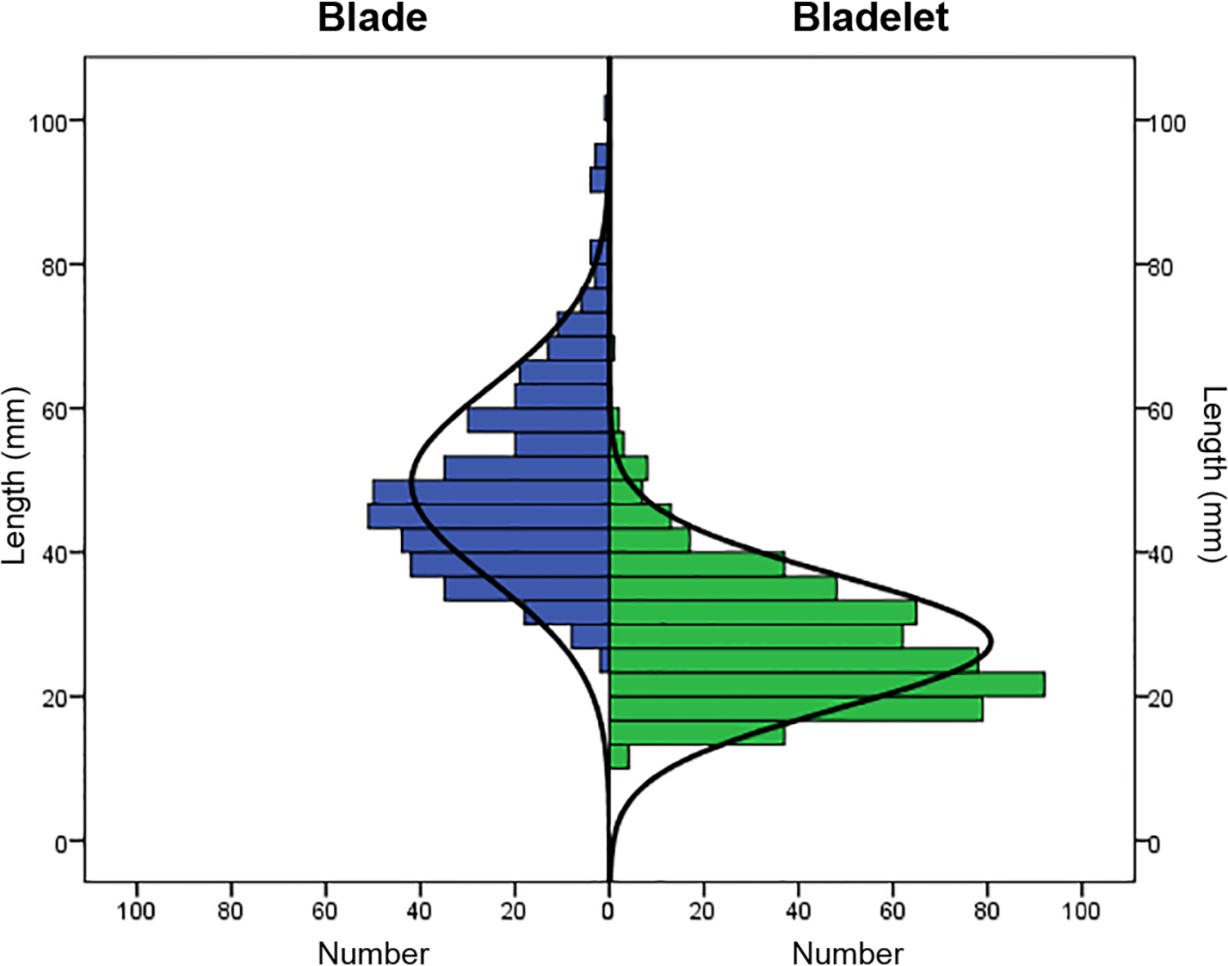
Comparison between the distribution of complete blade lengths (in millimeters; blue) and complete bladelet lengths (in millimeters; green).

When considered as a whole, the distribution of width measurements is unimodal (Fig 6). The median value falls in the bladelet range. Blade and bladelet length ranges overlap extensively (Fig 7), although the two categories have different medians (Mann–Whitney, U = 16691; p<0.01). Considered together, the length of elongated blanks in the seventy fifth percentile is 46.5 mm. Similar to length, blade and bladelet thickness ranges partially overlap (S3 Fig). Most of the blades are relatively small in sizes, even if the production of large-sized blades is evident by isolating the raw material unit (RMU [100]) of Oolithic flint. This was verified statistically using a series of Mann–Whitney tests comparing between blades made from Oolithic flint and all other blades together (S2 Table). Blades made from this coarse-grained flint are bigger in length (Mann–Whitney, U = 75; p<0.01), width (Mann–Whitney, U = 12479; p<0.01), and thickness (Mann–Whitney, U = 18519; p<0.01).

Concerning the width to thickness ratio, blade (4.3 ± 1.6 mm) and bladelet (4.2 ± 1.6 mm) means are not different (Mann–Whitney, U = 1.4E06, p = 0.7), indicating a constant robustness across blanks. The elongation ratio (length to width), instead, suggests a production of slender bladelets. The elongation mean for blades is 3.0 ± 0.6 mm, while for bladelets it is 3.4 ± 0.9 mm (Mann–Whitney, U = 82941, p<0.01).

#### Flakes

Flake morphological and technological attributes are listed in Table 4. The analysis of core reduction has already shown that flakes were not the main goal of lithic production. Flakes were mostly involved in the initialization and maintenance of blade and bladelet cores. Most of the flakes, however, have undiagnostic features that do not allow them to be placed in an unequivocal stage of the reduction sequence. Straight and slightly curved profiles dominate the assemblage. Certain types of cross-sections, less frequent across blades and bladelets, are common in the flake assemblage. This is especially true of flat and rectangular cross-sections. Dorsal scars attest to the application of unidirectional patterns, usually sub-parallel. The crossed scar pattern is, however, more common than in the previous categories and is frequently associated with semi-cortical flakes involved in the raw material decortication. Outline morphology and distal end attributes demonstrate that regular flakes were not the objective of the knapping. Most of them are, indeed, irregular and have stepped or plunging distal ends.

Finally, it must be mentioned that a small sample of flakes (n = 22), sometimes patinated, characterized by a high degree of predetermination and with faceted platforms has been identified. These flakes are technologically comparable to the Levallois unidirectional flakes found in the Final Mousterian layers [73]. Furthermore, flakes with centripetal scar patterns (n = 45) could be ascribed to the parallel core method previously described. Both groups are likely to represent the results of post-depositional events that marginally affected the integrity of the Protoaurignacian rather than to independent reduction sequences.

### Core initialization and maintenance interventions

This section aims to isolate and describe blanks that had a key role in the beginning of the reduction sequence of platform cores, but also in its progression. The information gained through the diacritic analyses of the initial and exhausted cores allowed us to identify the functions of certain by-products frequently obtained during the platform reduction methods. The description of these products is therefore closely related and dependent on the core analysis.

#### Initialization

**Fig 8:**
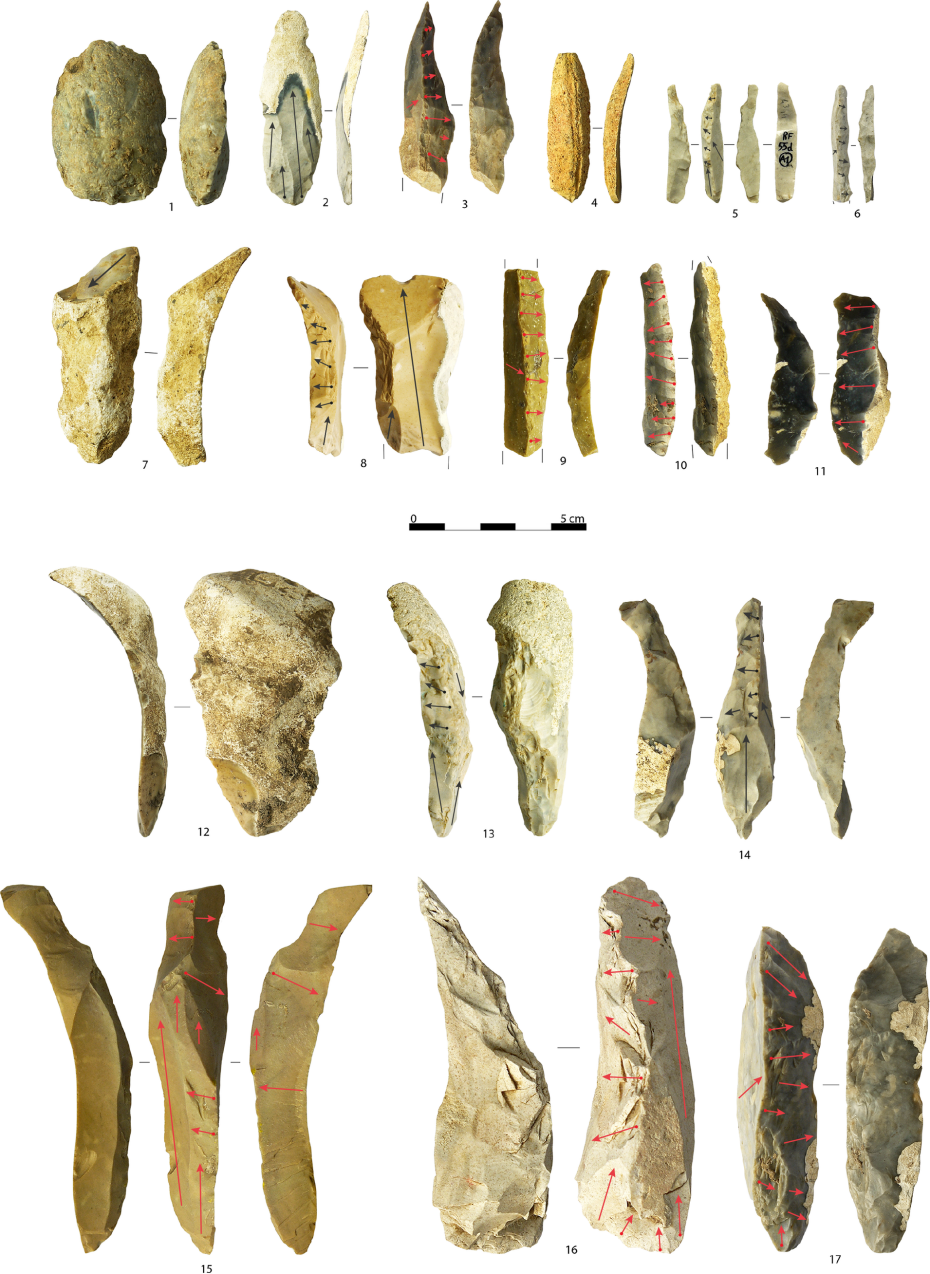
Blanks belonging to the decortication and initialization of platform cores. Fully cortical flakes (1, 12), semi-cortical blade with multiple bladelet scars (2), crested blades (3, 9, 11, 13–14, 17), fully cortical bladelet (4), crested bladelets displaying remains of the ventral face of the core blank (5, 6), fully cortical blade (7), crested flake (8), crested bladelet (10), naturally backed blade with the rest of a two-sided crest in the distal side (15), two-sided crested blade (16). Arrows indicate the direction of removals. Artifacts are oriented with the butt at the bottom of the photo (photo: A. Falcucci).

Fully cortical blades with steep triangular cross-sections attest to the frequent use of natural ridges present on the raw material nodules to start the blank production (Fig 8: 7). A favorable angle was usually found at the intersection of two faces. When the core blank was a flake, or was previously decorticated, initial blades bear cortical remains that usually range from 66% to 99%. The length of complete fully cortical blades (n = 7) and almost completely cortical blades (n = 14) ranges from 31.6 to 85.1 mm (mean: 55.0 mm). Given the small size of some products, these are at times likely to be part of bladelet core initialization (Fig 8: 2). Sometimes prior interventions to design the core volume structure was required. In these cases, the resulting products are both crested blades and two-sided crested blades (Fig 8: 15,16). Two-sided crests are less common and usually have a crested edge more developed than the other. Removals always come from the anterior side of the core, towards the flanks. Complete two-sided crested blade (n = 5) length ranges from 59.3 to 102.5 mm (mean: 70.0 mm). Crested blades are more common and were usually applied on smaller nodules. The crest could be produced starting from a cortical edge (Fig 8: 11,13), or at the junction with a perpendicular plain face (Fig 8: 3,14,17). In most cases, crests were performed only after a cortical blade or cortical flake was removed following the longitudinal axis of the flaking surface (n = 14; Fig 8: 9). Some of these share certain similarities with neo-crested blades, which are instead removed during the core maintenance operations, and may even be confused with them. Crests are usually continuous, even if removals are more pronounced in the mesio-distal side. Complete crested blade (n = 12) length ranges from 35.4 to 87.5 mm (mean: 56.9 mm). Some of these products are also likely to represent the first stage of bladelet core configuration. Secondary crested blades are not frequent, as crest removals were rather short and modified only a limited area of the core.

Fully cortical bladelets (Fig 8: 4) are less common, indicating that bladelet core initialization usually started with the removal of small blades. Crested bladelets are well represented in the assemblage, while two-sided crests are rare. As for blades, crest removals were shaped from the anterior side of the core towards the flanks and were more invasive starting from the medial part. In fourteen cases (38.9%), the opposite side of the crest displays remains of the ventral face of the core blank (Fig 8: 5,6). These artifacts belong to narrow-sided cores made from flake. They indicate that crests were performed at the junction of the ventral face with the dorsal side, along the longitudinal axis. Crested bladelets also attest to the selection of small nodules (n = 2) and the recycling of previous cores to pursue the production of lamellar blanks (n = 3). In these cases, the perpendicular laminar removals of the previous reduction stage act as crests [101]. Complete crested bladelets (n = 8) length ranges from 18.8 to 50.0 mm (mean: 30.4 mm) and, except in the case of the longer specimen, do not exceed 33.0 mm in length. Thus, they were applied on relative small cores.

Flakes were frequently used to partially decorticate the raw material nodules (Fig 8: 12). A frequent operation consisted of the removal of a thick cortical flake to create a flat striking platform (Fig 8: 1). Flakes were also used to allow the first laminar negative to be detached, sometimes opening temporary striking platforms to shape an opposite crest. Crested flakes (Fig 8: 8) are not common and have lengths that range from 25.0 to 95.0 mm (mean: 50.0 mm).

#### Maintenance

**Fig 9:**
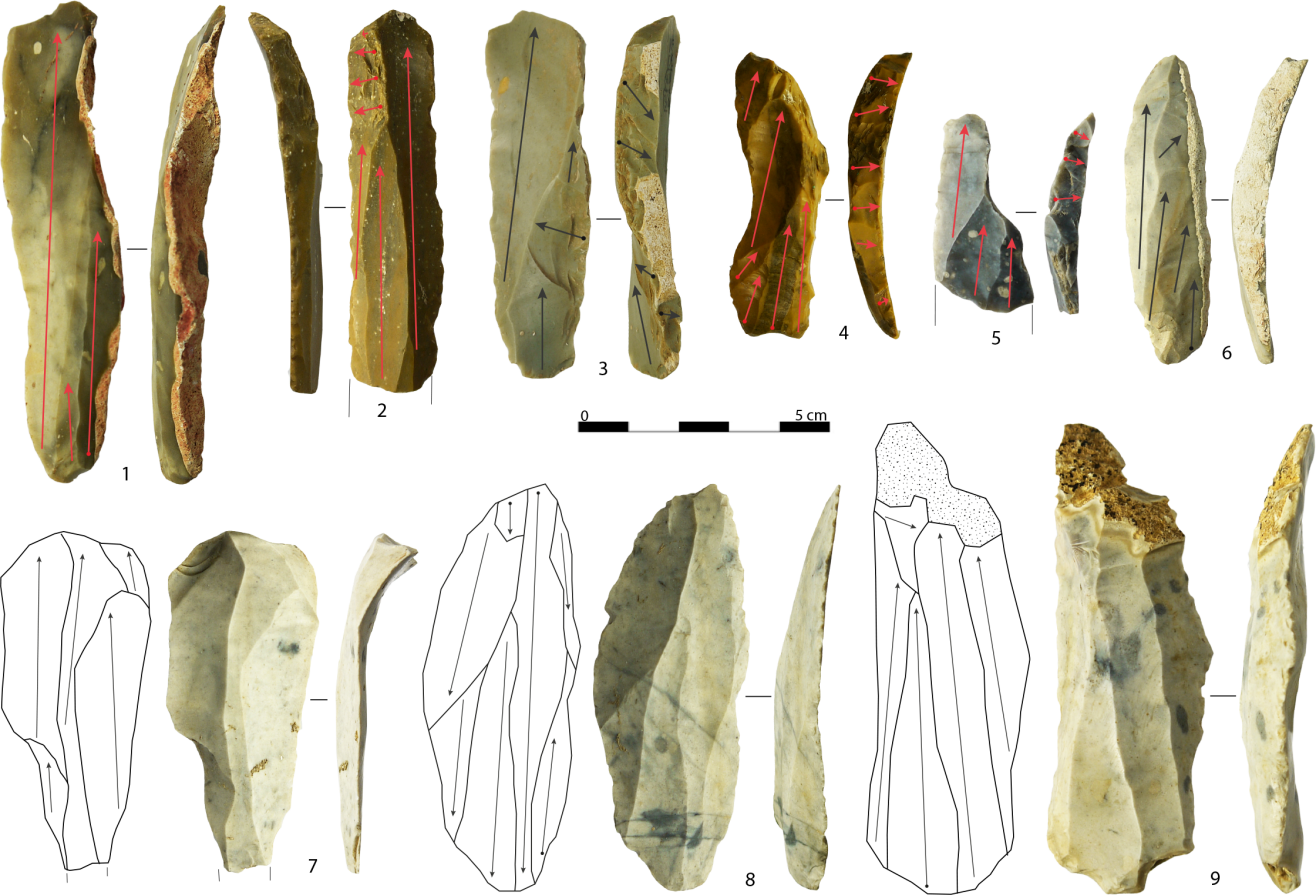
Maintenance products from blade production. Naturally backed blades (1, 6), neo-crested blades (2–5), and technical blades with multiple blade scars (7–9). Arrows indicate the direction of removals. Artifacts are oriented with the butt at the bottom of the photo (photo and drawings: A. Falcucci).

Maintenance products are common among blades. Their function was to maintain and re-establish the lateral and longitudinal convexities of the core, but also to rejuvenate part of the flaking surface. The most common operations carried out on blade cores resulted in naturally backed blades (Fig 9: 1, 6) and neo-crested blades (Fig 9: 2–5). Both products are commonly related to a sub-parallel reduction pattern and aimed to control the lateral convexities of the core during a continuous linear progression that alternates detachments at the center of the flaking surface and at the intersection with a perpendicular core side [94]. Naturally backed blades are an expression of the opportunistic exploitation of available edges, while neo-crested blades reveal a major technical investment. Neo-crested blades usually display a backed edge. Neo-crest removals are, in most cases, located on the mesio-distal side of the core and, in only seven cases (13.7%), invade the whole length of the blank.

**Fig 10:**
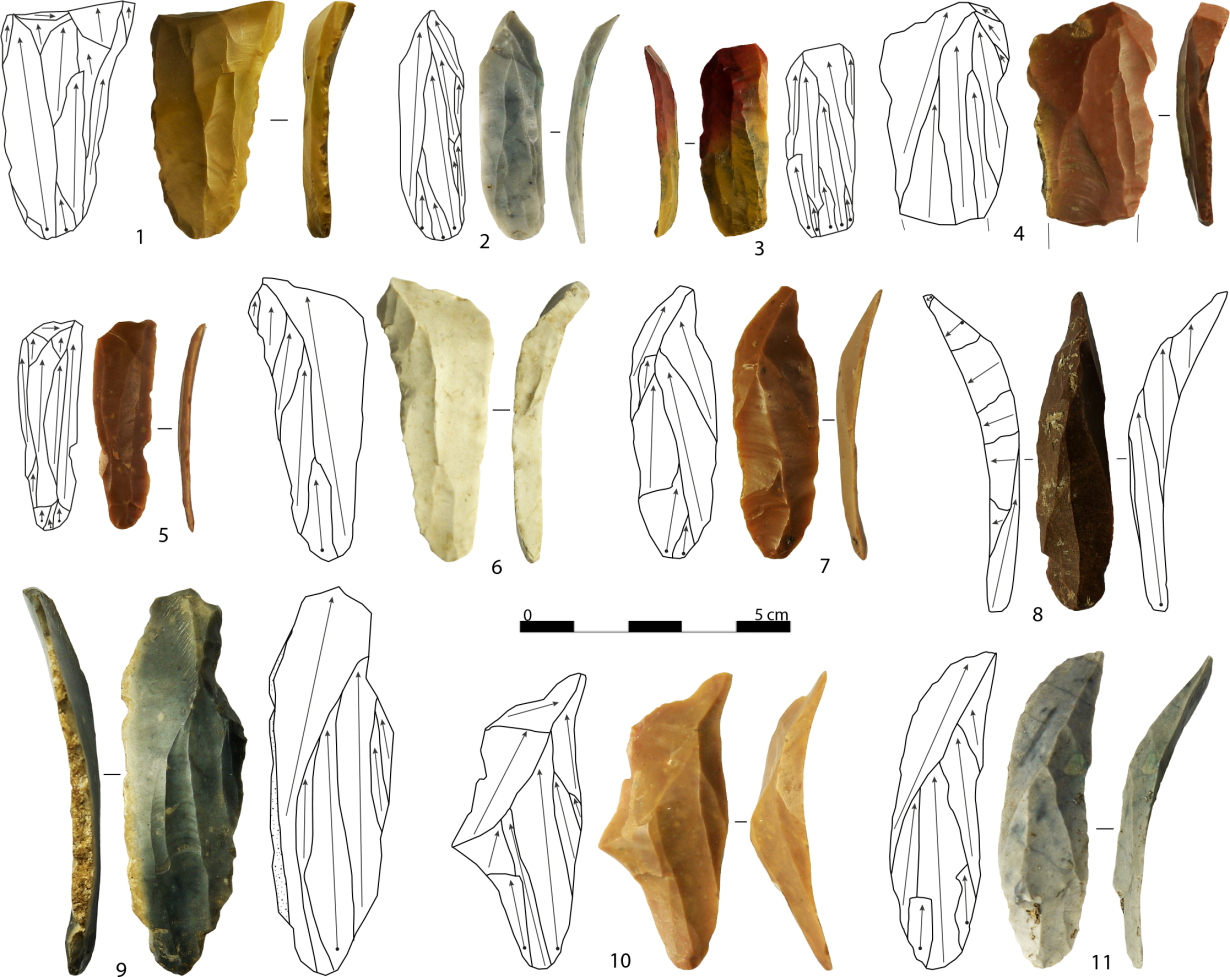
Maintenance products from bladelet and simultaneous blade-bladelet productions. Technical blades with multiple bladelet scars (1–5, 9), lateral comma-like blades with multiple bladelet scars (6–8, 10, 11). Arrows indicate the direction of removals. Artifacts are oriented with the butt at the bottom of the photo (photo and drawings: A. Falcucci).

The technical blade category includes all by-products detached at the center of the flaking surface with the aim to remove critical parts of the core or to accentuate the distal core convexity (Fig 9: 7–9; Fig 10: 1–5, 9). For these reasons, they are characterized by polyhedral cross-sections (65%) and plunging (51%) or stepped (14.6%) distal ends. The most striking feature of technical blades is that they have in eighty-six cases (73.5%) from one to seven bladelet negatives on their dorsal face (Fig 10: 1–5, 9). Even if they correspond to cores characterized by a simultaneous production of small blades and big bladelets in few cases, most of them correspond to maintenance operations carried out on bladelet cores. A plunging technical blade refitted to a semi-circumferential bladelet core (Fig 2: 8) is a good example of this operation.

The last category of blade maintenance products was named lateral comma-like blade after Porraz et al. [102] (Fig 10: 6–8, 10, 11). Lateral comma-like blades represent the most frequent maintenance operation carried out at the junction of core faces during convergent reduction patterns that target pointed bladelets, but also during the shaping of initial blade or bladelet cores in order to isolate the future flaking surface. Lateral comma-like blades have distal ends with an off-axis orientation and usually have asymmetrical cross sections (55.4%) and a twisted (50.6%) or intense curved (21.7%) profile. Distal ends are usually plunging (57.9%) or stepped (13.2%), as they remove part of the core base. As for technical blades, they usually display lamellar negatives on the dorsal face (54.2%).

The study of blades displaying lamellar negatives was highly informative. The number of these products among the studied sample is considerable (n = 265, MNFP = 198). The fact that many of those blades have been interpreted as by-products of the lamellar production system suggests that a remarkable amount of blades was not the primary intention of blank production, instead, it was part of elaborate maintenance operations carried out on bladelet cores. Complete blades with lamellar dorsal negatives (n = 121) have lengths ranging from 26.4 to 75.6 mm (mean: 46.4 mm; median: 45.4 mm). They are, indeed, significantly shorter than the rest of the analyzed blades (Mann–Whitney, U = 17209; p<0.01).

It has been shown that all range of maintenance operations on bladelet cores were usually performed by blades. For this reason, maintenance products on bladelets are low in frequency. Neo-crested bladelets are not common. They have asymmetrical cross-sections and in most cases a sub-parallel dorsal scars pattern (76.5%). Technical bladelets and lateral comma-like bladelets do not differ from the same products made from blades. Both products display regular lamellar negatives on dorsal sides, usually belonging to short, pointed bladelets.

Partial and total core tablets were frequently used to manage the striking platform. Table 6 lists relevant attributes detected on these by-products.

**Table 6:** List of relevant attributes recorded on core tablets.

Core tablet attributes	
Knapping progression	
Frontal, narrow face	33 (20.1%)
Frontal, wide face	21 (12.8%)
Semi-circumferential	90 (54.9%)
Undetermined	20 (12.2%)
Blank production	
Blade	25 (15.2%)
Bladelet	115 (70.1%)
Blade-bladelet	24 (14.6%)
Core flaking surface width	
Blade core	46.7 ± 10.4
Bladelet core	27.3 ± 6.1
Blade-bladelet core	37.0 ± 13.4

Core flaking surface width was measurable only on total core tablets (n = 67). Percentages are given in brackets.

**Fig 11:**
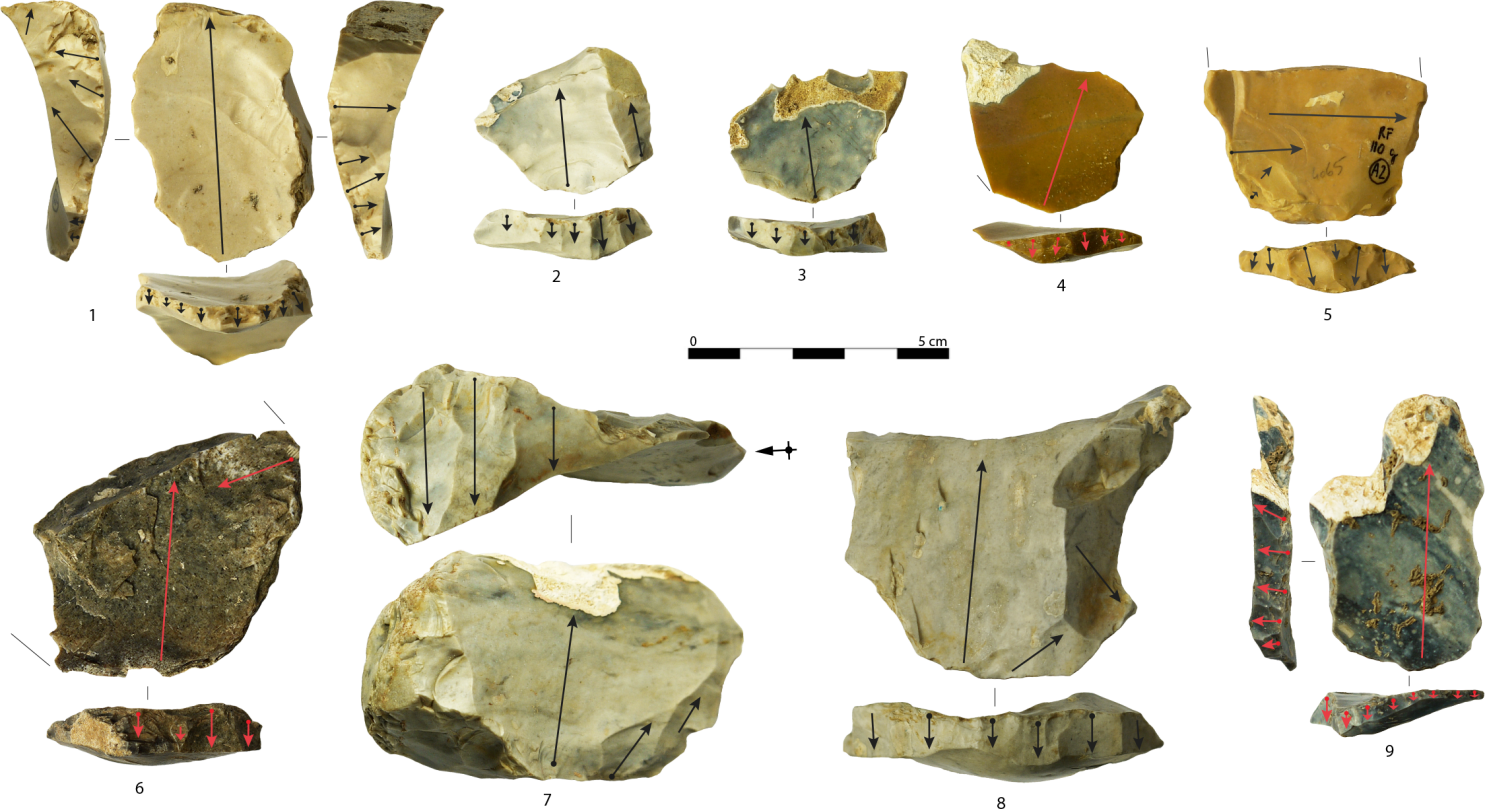
Core tablets. Blade core tablets (6–8) and bladelet core tablets (1–5, 9). Arrows indicate the direction of the blow and of removals (photo: A. Falcucci).

They are clearly linked to the identified core types. As expected, most of them belong to bladelet cores. Total core tablets (n = 67) allow us to measure the width of the related core flaking surface. Blade core tablets (Fig 11: 6–8) display broader flaking surfaces compared to blade-bladelet or bladelet cores (Fig 11: 1–5, 9). Among blade core tablets, large-sized cores were identified. They may have been highly reduced on site or exported. The latter case is exemplified by a core tablet on Oolithic flint (Fig 11: 6) that is associated with several blades and whose discarded core has not been found.

**Fig 12:**
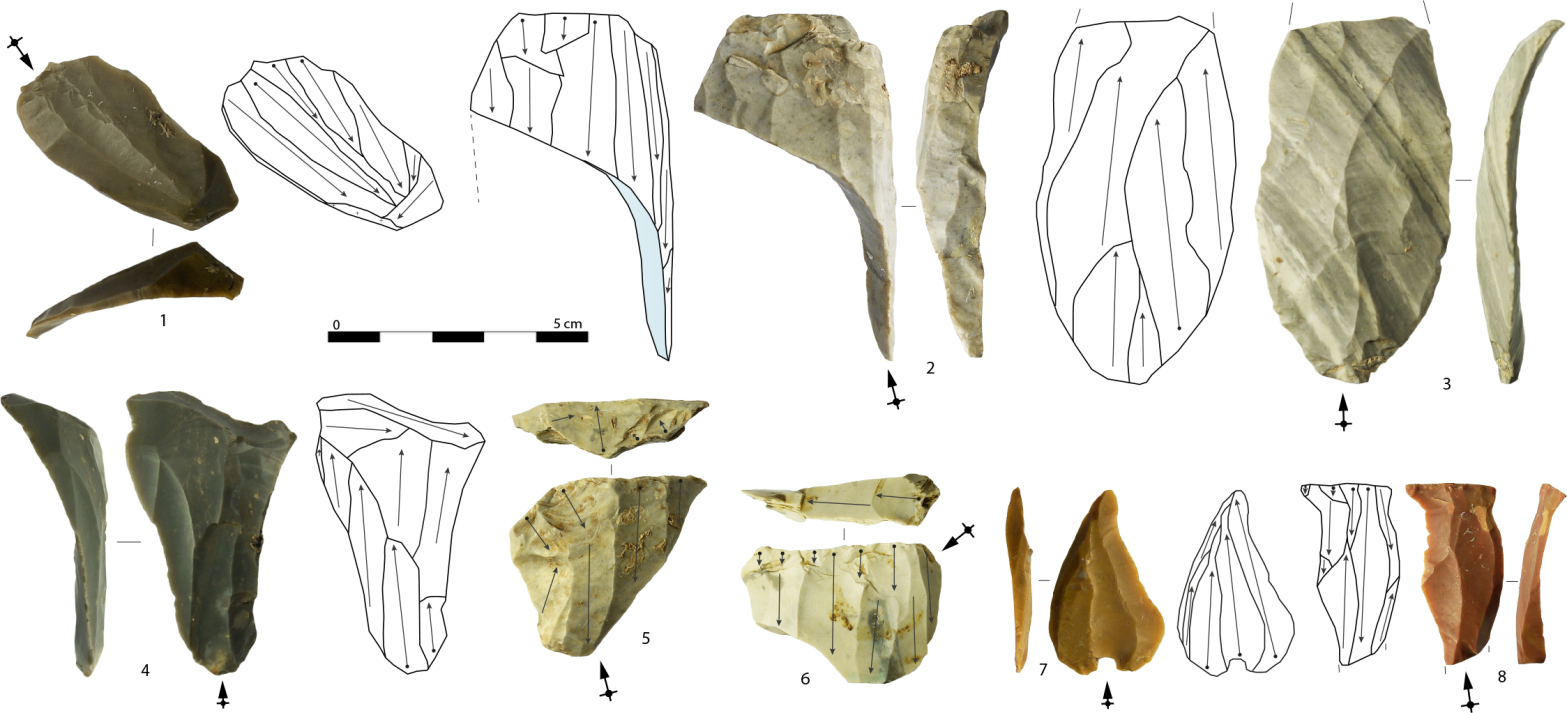
Technical flakes. Technical flakes removed from bladelet cores (1, 5–8), blade cores (2, 3), and blade-bladelet cores (4). Note that 2 is a spall removed from a technical flake. Arrows indicate the direction of the blow and of removals (photo and drawings: A. Falcucci).

Technical flakes are another important source of information because they display evidence of laminar and lamellar production at different reduction stages (Fig 12). Sometimes technical flakes rejuvenated most of the flaking surface prior, or slightly after, the core rotation (Fig 12: 8). Technical flakes display up to eight blade or bladelet negatives. Last visible negatives allow us to link some of them to a blade production (n = 33, 22.1%), others to a simultaneous blade-bladelet production (n = 15, 10.1%), and finally to a bladelet production (n = 86, 57.7%). The remaining products are unidentifiable (n = 15, 10.1%). The length of complete technical flakes (n = 87) ranges from 10.9 to 116.0 mm (mean: 42.2 mm). Technical flakes with blade scars belong to cores of different sizes and display blades with lengths ranging from 39.0 to 95.2 mm. A Kruskall–Wallis test was run to evaluate the differences among complete technical flakes with laminar, lamellar, and simultaneous negatives (H = 15.63, p<0.01). Flakes with bladelet negatives are smaller than the others, while flakes with a simultaneous blade-bladelet production are not different from flakes with blade negatives (S3 Table). This evidence indicates that simultaneous blade-bladelet productions were carried out from the initial stages of core exploitation.

Neo-crested flakes and lateral comma-like flakes are less common than in the blade and bladelet categories. In most cases, they manifest a failed attempt to remove a laminar blank.

### Tools

Table 7 gives a general overview of the main tool categories. This section does not aim to describe retouched tools from a typological perspective, but instead seeks to identify signatures relevant for the technological analysis.

**Table 7:** General overview of the main tool categories.

Tool categories	Number	MNFP
Retouched bladelet	2481 (78.1%)	912 (69.6%)
Retouched blade	239 (7.5%)	130 (9.9%)
Retouched flake	98 (3.1%)	66 (5%)
Retouch, undetermined	4 (0.1%)	-
Burin	104 (3.3%)	63 (4.8%)
Burin + lateral retouch	16 (0.5%)	10 (0.8%)
End-scraper	107 (3.4%)	61 (4.7%)
End-scraper + burin	4 (0.1%)	1 (0.1%)
End-scraper + lateral retouch	18 (0.6%)	13 (1%)
End-scraper + truncation	1 (-)	1 (0.1%)
End-scraper + splintered piece	1 (-)	1 (0,1%)
Truncation	34 (1.1%)	20 (1.5%)
Truncation + lateral retouch	25 (0.8%)	10 (0.8%)
Splintered piece	45 (1.4%)	22 (1.7%)
Total	3177 (100%)	1310 (100%)
**Blank types**		
Bladelet	2514 (79.1%)	927 (70.8%)
Blade	424 (13.3%)	229 (17.5%)
Flake	222 (7%)	150 (11.5%)
Undetermined	17 (0.5%)	4 (0.3%)

Percentages are given in brackets.

**Fig 13:**
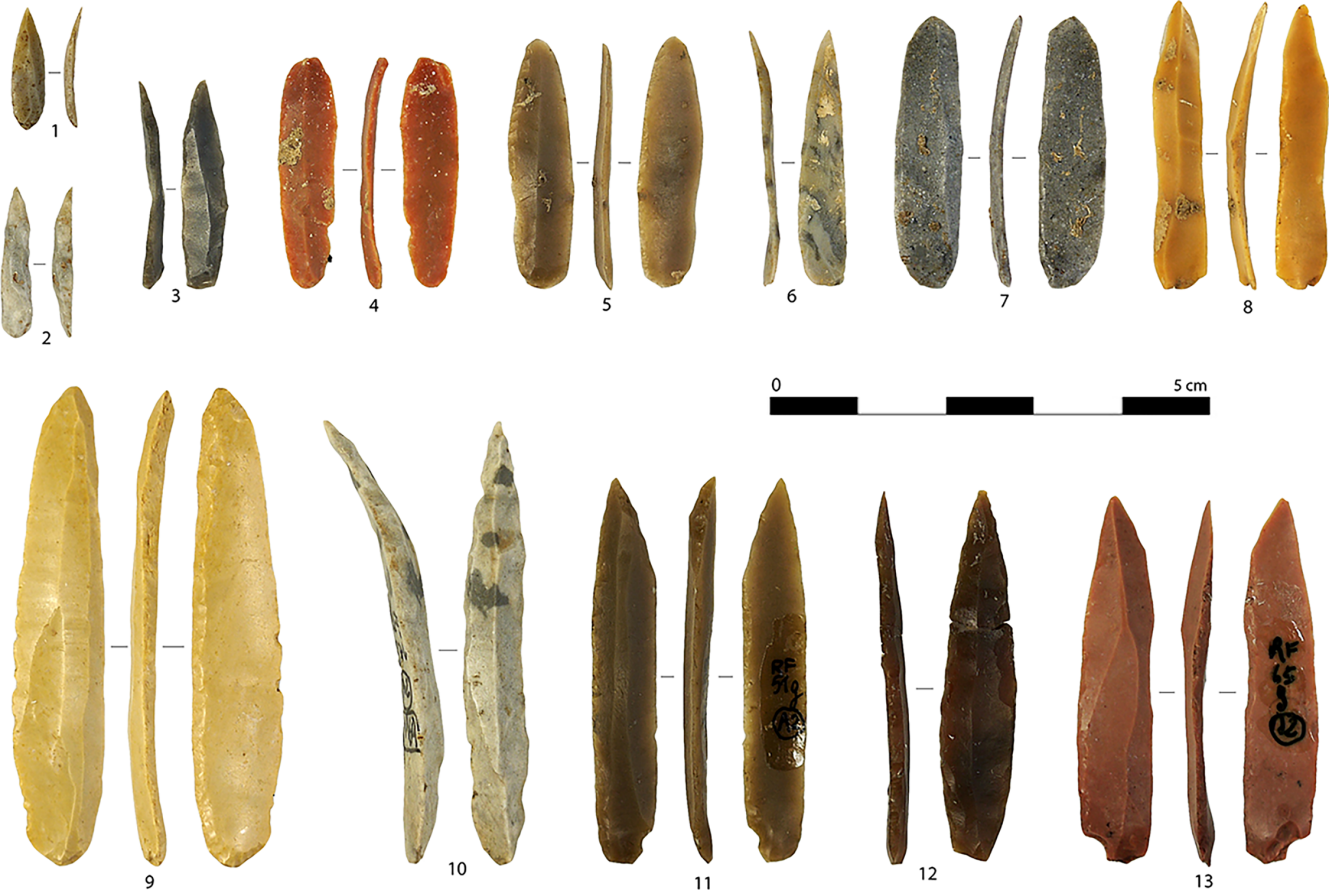
Retouched bladelets with convergent (1–3, 6, 10–13) and lateral (4–5, 7–9) retouch (typological definition after Falcucci et al. [103]). Retouching is direct on 1–3, 6, 10, and 12; alternate on 4–5, 11, and 13; inverse on 7–9 (photo: A. Falcucci).

The most striking feature of the assemblage is the dominance of tools made from bladelets. Retouched bladelets represent 26% (MNFP = 20.5%) of the whole bladelet assemblage. This index is very low for blades (7%, MNFP = 7.4%) and especially flakes (2.4%, MNFP = 3.2%). Tools on bladelets represent a rather homogeneous category. They are, in most cases, only modified on the edges by applying a marginal retouch and have been typed as bladelet with lateral retouch (Fig 13: 4–9) and bladelet with convergent retouch (Fig 13: 1–3, 10–13) according to the external blank morphology [103].

**Fig 14:**
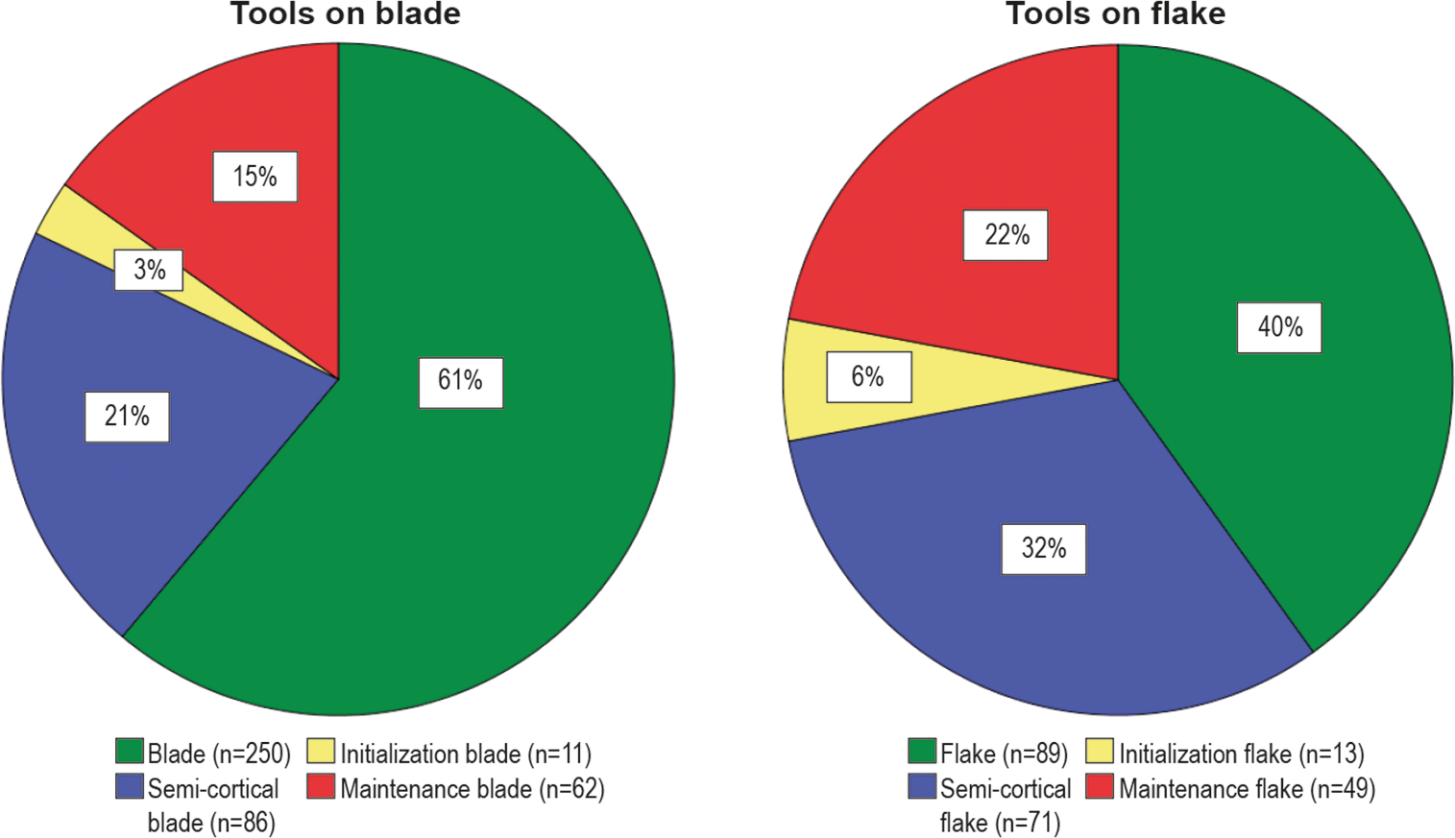
Pie charts representing the proportion of tools made on blades (left) and flakes (right), grouped according to the main technological categories. Initialization group includes fully cortical and crested elements; maintenance group includes crested secondary, naturally backed, neo-crested, lateral comma-like, and technical blanks. For colors see the legend.

**Fig 15:**
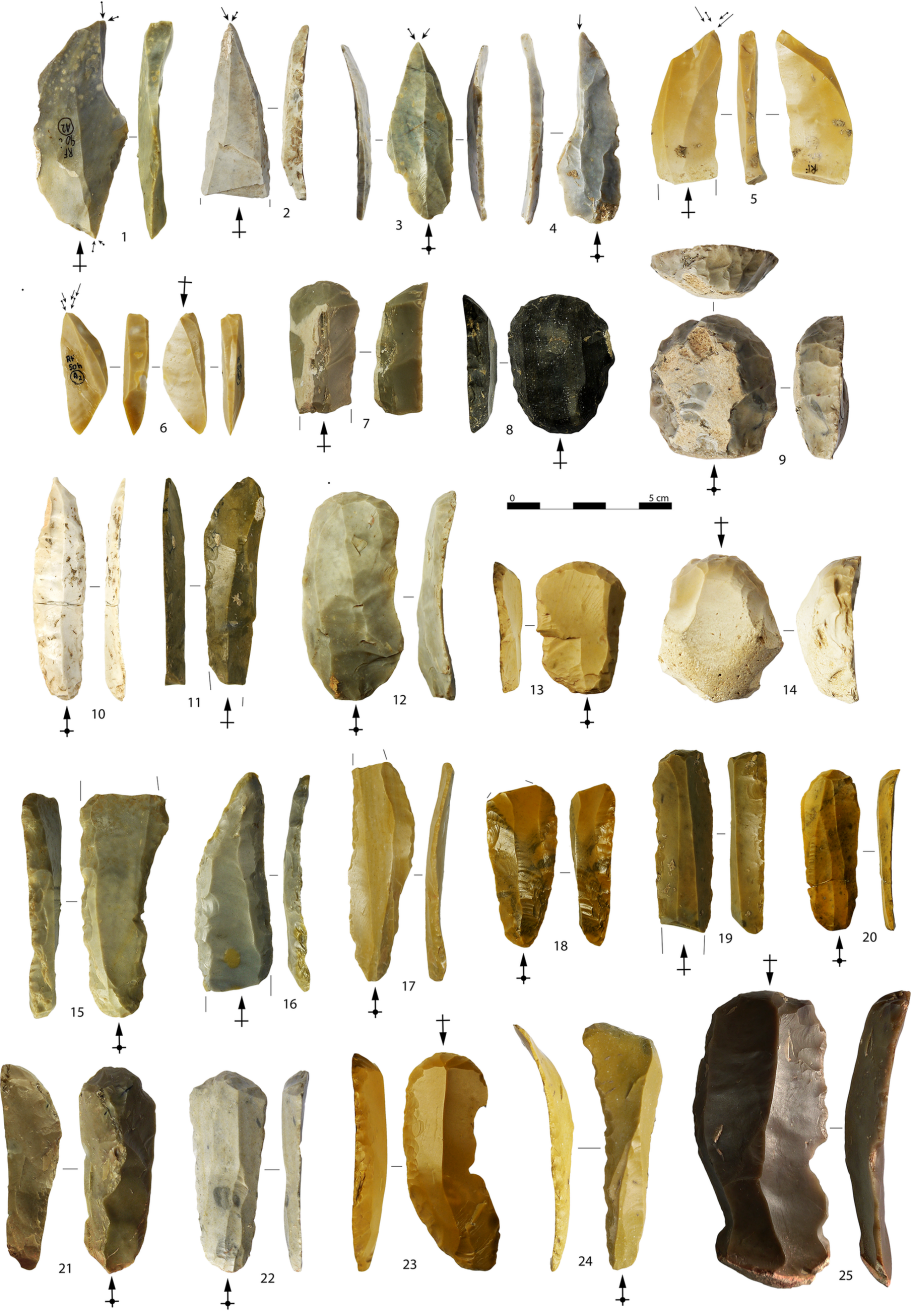
Examples of tools. Burins on blade (1–6), end-scrapers on crested blades (7, 21), end-scrapers on flake (8, 13), thick end-scrapers on cortical flakes (9, 14), blades with lateral retouch (10–11, 17–19, 24), end-scraper on a technical flake with blade scars (12), thick blades with Aurignacian retouch (15, 16), end-scraper on a technical blade with bladelet scars (20), end-scraper on blade (22) belonging to the first reduction phase of core number 5 in Fig 2, end-scrapers with lateral scalar retouch on blades (23, 25). Arrows indicate the direction of the blow (photo: A. Falcucci).

Retouched bladelets have regular outline morphologies and almost always lack cortical remains (98.7%). On the contrary, cortical remains are frequently found on tools on blades (29.5%), and especially tools on flakes (49.1%). Bladelet tools have been manufactured from by-products of the core reduction sequence only in two cases. This data is different for blades and flakes, as the selection of by-products is relatively high (Figs 14 and 15).

Among blade tools, fifty-three pieces (12.5%) display lamellar negatives on the dorsal side. This evidence suggests that, along with blanks coming from a proper blade production, some blanks could be selected among the waste of bladelet reduction strategies. Common tools are dominated by laterally-retouched blades (Fig 15: 10–11, 15–19, 24) followed by end-scrapers (Fig 15: 7–9, 12–14, 20–23, 25), and burins (Fig 15: 1–6). Six blades display intense scalar retouching and can be classified as Aurignacian blades (Fig 15: 15–16). They may be correlated to a protracted use and to a possible introduction of formal tools.

Table 8 shows metric comparisons between blanks and tools according to the blank category and the results of multiple Mann–Whitney tests. The bigger blade and flake products were systematically selected. For bladelet tools the opposite can be said; they have inferior width and thickness values, but differences in length are not significant. The relatively high difference in width may be explained in part as a selection of the narrower products, but mostly as a consequence of retouching.

**Table 8:** Metrical comparison of the mean values (in millimeters) ± standard deviations between tools and blanks according to the main blank types, and results of the multiple Mann–Whitney U-tests (*p* values) that were conducted.

	Blade			Bladelet			Flake		
	Blank	Tool	p-value	Blank	Tool	p-value	Blank	Tool	p-value
**Length**	49.6±13.3	60.5±18.3	p<0.01	27.6±9.11	28.3±8.8	p = 0.25	37.1±13.2	43.8±14.4	p<0.01
**Width**	16.5±4.0	19.5±5.5	p<0.01	8.8±2.0	6.6±1.8	p<0.01	25.2±9.6	30.3±9.4	p<0.01
**Thickness**	4.5±2.2	5.9±2.5	p<0.01	2.4±1.1	1.7±0.6	p<0.01	6.8±4.0	10.1±4.4	p<0.01

### Knapping technique

Table 9 gives an overview of the criteria that have been used to identify the knapping techniques. All features agree with a direct application of force. Differences can be found in the gesture involved in the detachment of blades, bladelets, and flakes. For blades and bladelets, the high frequency of dorsal thinning to reduce the overhang, the small thickness of platforms, the presence of lips, and the EPA values clearly indicates a marginal percussion. However, some blades were knapped with an internal striking gesture. This was detected by the higher frequency of bulbs and a certain number of thicker platforms, especially among blades involved in core maintenance operations.

**Table 9:** List of the attributes used to identify the knapping technique.

Knapping technique	Blade	Bladelet	Flake
Platform measurements			
Width	4.2±2.4	2.4±1.2	8.8±6.2
Thickness	1.6±1.1	0.8±0.5	3.4±2.7
Ratio W/T	3.2±2.5	4.1±4.2	3.3±3.3
EPA			
≤ 45°	83 (6.7%)	59 (2.8%)	63 (6.6%)
≤ 60°	443 (35.5%)	726 (34.2%)	234 (24.5%)
≤ 75°	613 (49.2%)	1271 (60%)	455 (47.7%)
≤ 90°	66 (5.3%)	19 (0.9%)	153 (16%)
Undetermined	42 (3.4%)	45 (2.1%)	49 (5.1%)
Platform type			
Plain	923 (74%)	1299 (61.3%)	596 (62.5%)
Linear	138 (11.1%)	543 (25.6%)	48 (5.0%)
Punctiform	36 (2.9%)	166 (7.8%)	13 (1.3%)
Faceted	21 (1.7%)	1 (.0%)	86 (9.0%)
Other	129 (10.3%)	111 (5.3%)	211 (22.1%)
Dorsal thinning			
Yes	1049 (84.1%)	1931 (91.1%)	398 (41.7%)
No	154 (12.3%)	147 (6.9%)	509 (53.4%)
Undetermined	44 (3.5%)	42 (2%)	47 (4.9%)
Bulb			
Yes, moderate	495 (39.7%)	569 (26.8%)	432 (45.3%)
Yes, pronounced	51 (4.1%)	18 (0.8%)	135 (14.2%)
No	659 (52.8%)	1491 (70.3%)	339 (35.5%)
Undetermined	42 (3.4%)	42 (2%)	48 (5%)
Lip			
Yes, moderate	477 (38.3%)	1074 (50.7%)	208 (21.8%)
Yes, pronounced	642 (51.5%)	921 (43.4%)	336 (35.2%)
No	86 (6.9%)	83 (3.9%)	362 (37.9%)
Undetermined	42 (3.4%)	42 (2%)	48 (5%)
Bulbar scars			
Yes	257 (20.6%)	197 (9.3%)	246 (25.8%)
No	948 (76%)	1881 (88.7%)	660 (69.2%)
Undetermined	42 (3.4%)	42 (2%)	48 (5%)

EPA: external platform angle. Percentages are given in brackets.

Flake platforms are very similar to blade and bladelet platforms, with most of them being plain. However, they are characterized by a combination of features that can be explained as an ambivalence of striking gestures that involved both marginal and internal percussion. Internal percussion is evident in the presence of thick platforms, some of them above the 4 mm border suggested by Pelegrin [104]. The lower frequency of dorsal thinning and lips, the higher frequency of pronounced bulbs, and the higher EPA values compared to laminar blanks argue in favor of this hypothesis. It is worth mentioning a small sample of flakes characterized by facetted platforms. As previously said, they are frequently found in flakes that are technologically very different from the rest of the assemblage. Their frequency is, however, very low and does not affect the general reconstruction of knapping techniques across flakes. To conclude, flakes were produced both with internal and marginal percussion at different stages of the reduction sequence.

The type of knapping tool involved in lithic production for this assemblage will not be directly addressed, following recent experimental works that have criticized the unequivocal distinction between the use of hard or soft stone and organic hammers [105–107]. However, it can be noted that there is a relatively high frequency of bulbar scars (esquillement bulbaire [85]) especially among blades and flakes. Bulbar scars are sometimes associated with fine ripples in the first millimeters of the ventral face. This evidence, together with the frequent association of lips and moderate bulbs, suggests that soft stone hammers were part of the involved knapping tools [95], which should be confirmed from the use-wear traces observed on most of the stone hammers in the course of examination.

## Discussion

### The issue of the continuous reduction sequence

The extensive analysis conducted on the Protoaurignacian of Fumane Cave permits us to carefully address the technological definition of this techno-complex. Before discussing its internal and geographical variability, a critical review of the so-called continuous reduction sequence [35, 47, 48, 51] is needed. Based on the results of this study, it can be underlined that bladelets do not originate from reduced blade cores. Independent and variable reduction strategies are common at Fumane and, more generally, in the Protoaurignacian assemblages of Mochi and Bombrini [24, 43, 61], La Fabbrica [108], Castelcivita [109], Observatoire [110], Mandrin [111, 112], Esquicho-Grapaou [113], Arbreda [114], Labeko Koba [56], La Viña [37], Isturitz [66], Arcy [49, 115], Româneşti and Tincova [65, 116], and Siuren I [117, 118].

Given the absence of extensive refitting analyses, the assumption that bladelets were the result of decreasing core size is supported by three main arguments: the absence of blade cores, the morphological affinity between blades and bladelets, and, finally, the dimensional continuity between them [37, 38, 49, 51, 119–121]. Our results disagree with these points.

First, blade cores have been found at Fumane, Bombrini [43], Româneşti and Tincova [65, 116], Mandrin [111], Arbreda [114], La Viña [37], Piage [47], and Les Cottés [122]. They are generally reduced, but the last complete negatives correspond to blades. At Les Cottés fifteen blade cores (32% of the core collection) were found; a frequency that is even higher when compared to the upper Early Aurignacian layer [122]. At Fumane and Arbreda [114], blade cores or blade core fragments could be recycled into bladelet cores, which implied a general reorganization of their structure. This is also the case in the Early Aurignacian of Geißenklösterle, Champ-Parel and Hui [51, 123–125]. At Fumane and Labeko Koba [56], non-exhausted blade cores were likely exported, while at Mochi and Bombrini, blades made from high-quality raw material nodules were knapped elsewhere and imported as formal tools [43]. The same has been proposed for some large-sized blades found at Mandrin [112], Arcy [126], and Kozarnika [120]. It is worth mentioning that the techno-economic dissociation of blade and bladelet reduction strategies over a large territory is a feature commonly associated with the Early Aurignacian [54, 127]. This behavior reflects constraints in raw material availability in certain regions. While at Fumane, large-sized nodules could be found within few kilometers from the site [83], at Bombrini and Mochi human groups often had to rely upon extra-local flint coming from the French Provence or the Italian Apennines [128].

Second, blades and bladelets have indeed a certain affinity, noticeable in the preparation of flat striking platforms and in the systematic abrasion of the overhang related to the use of direct marginal percussion. At Fumane, however, bladelets often have a convergent and pointed outline and are produced following a convergent reduction pattern. Blades are instead produced with sub-parallel reduction patterns, following procedures commonly described in Early Aurignacian assemblages [35].

Third, the dimensional overlap between blades and bladelets is not a reliable proxy to detect a continuous stone knapping sequence. This is indeed a pattern originating from the incorporation of products resulting from different temporal events into a unique and, apparently, linear distribution. According to the initial volume of the raw material nodule, the first stage of bladelet core reduction could sometimes result in the extraction of blade-sized blanks. The fact that the production tended rapidly to bladelets does not allow such evidence to speak for a continuous reduction process that started from large blade cores. In other words, bladelets were the objective of production before that first lamellar blank was detached, as also noticed by Bon [35] in one of the first description of the Protoaurignacian lithic technology. During the optimal phase of production, maintenance products, such as lateral comma-like blades and technical blades, could be intercalated to bladelets. They are shared elements in the Protoaurignacian and have been well described at Arcy [49], Esquicho-Grapaou and Louza [113, 129], Observatoire [110], and Kozarnika [120].

Blade and bladelet productions are not, however, always independent, as a simultaneous production of small blades and big bladelets has been demonstrated at Fumane, Labeko Koba [56], and Siuren I [118]. In all these cases, simultaneous production started from the early stage of core reduction, which is also one of the reasons for the overall dimensional continuity that exists between blades and bladelets.

To conclude, the most commonly used technological trait that is said to define the Protoaurignacian has been over-emphasized, and other features are needed to isolate its lithic technology.

### Protoaurignacian lithic technologies: Fumane in the European context

The most relevant features of the Protoaurignacian industry at Fumane Cave are the systematic and variable bladelet production and the dominance of retouched bladelets among tools. Most of the artifacts discarded at the site indeed belong to bladelets and by-products of lamellar reduction strategies. This is very different from the Uluzzian layers A4 and A3, in which bladelets played a minor role in the lithic system [15].

Bladelet-based industries mark the full consolidation of new technical solutions for the manufacture of small lithic implements, probably intended to be hafted in composite tools, at the beginning of the Eurasian Upper Paleolithic [55]. They are a shared feature of the Protoaurignacian across Europe, as evident at Fumane, Bombrini [43, 130], Mochi [61, 131], Observatoire [110], Esquicho-Grapaou [113, 132], Louza [129, 132], Mandrin [111, 112], Arbreda [114], Morín [119, 133], La Viña [37], Labeko Koba [38, 56], Isturitz [134, 135], Piage [36, 136], Les Cottés [122], Arcy [49, 115], Tincova [116, 137], Româneşti [65], Kozarnika [120], and Siuren I [64, 117, 138]. In these assemblages, bladelet production is characterized by a relatively broad range of core reduction strategies and is carried out on high quality raw material nodules. At Fumane, intact nodules and fragments were brought to the site where the future cores were roughly prepared. Non-invasive crests were applied only when the morphology of the blank did not permit the direct extraction of laminar products. According to the volume of the selected raw material nodule, bladelet core initialization could sometimes result in a first series of blade removals, as seen also at Observatoire [110]. In some cases, the most robust blanks produced in this initial reduction stage were selected to manufacture tools as end-scrapers, burins, and laterally-retouched blades and flakes. At Isturitz [66, 134] and Arcy [126] the selection of these by-products to manufacture tools is documented.

The optimal production phase took place on cores that were almost completely deprived of cortex and targeted bladelets of variable sizes. The frequent application of convergent and secondly sub-parallel reduction patterns resulted in the production of bladelets with pointed outlines, as well as bladelets with sub-parallel edges. Convergent reduction patterns are common in the entire extent of the Protoaurignacian and are associated with highly diagnostic maintenance operations such as lateral comma-like blades. These operations were usually carried out along the longitudinal axis of the flaked surface and in most cases from the main striking platform. At Fumane, the length of such products is compatible with most of the exhausted cores. Lateral comma-like blanks were detached at the intersection of core faces, isolating rather short surfaces and allowing the production of regular bladelets from early reduction phases [94]. The protracted alternation of primary blanks and by-products required the exploitation of most of the available surfaces by means of a semi-circumferential core progression. Most of these cores are usually classified sub-prismatic and sub-pyramidal cores and are found in all Protoaurignacian industries.

At Fumane, besides semi-circumferential cores, narrow-sided cores had a major importance and were exclusively used to produce bladelets. Narrow-sided cores were made from flakes and flat raw material nodules and targeted slender and rather straight bladelets. At Arbreda, they have served to produce small blades [114], while in other sites they are always described as bladelet cores. The initialization and maintenance operations carried out on narrow-sided cores at Observatoire [110] and Arcy [115] are comparable to Fumane. The production usually began with crested bladelets, well-represented in our studied assemblage, detached at the junction of the ventral face of the core blank. The extraction of regular bladelets was then achieved by lateral removals that converged towards the center of the flaking surface.

Core re-orientation was also a frequent strategy used to increase production efficiency. Multi-platform cores are frequent at Fumane and Mochi (40% of cores [61]) and are reported at Arcy [115], Isturitz [66], Arbreda [114], and Siuren I [118]. This evidence contradicts the assumption that core re-orientation is rare in the Protoaurignacian [139].

As showed, the flaking surface of bladelet cores was oriented, in most cases, according to the longitudinal axis of the blank, which represents one of the main technological features of the Protoaurignacian. Carinated technology is thus generally less well-represented compared to Early Aurignacian industries [35]. The technological organization of Protoaurignacian carinated cores, however, does not differ from the Early Aurignacian (as described in [35, 125]). Carinated cores are rare in the Ligurian region and in Southeast France [24, 43, 110, 132], but are the dominant bladelet production strategy at Arbreda [114] and are well-represented in northern Spain [37, 119], Pyrenean region [56, 66, 140], and Eastern Europe [65, 116, 118]. At Fumane, carinated cores do not differ much from semi-circumferential bladelet cores. The use of lateral removals to isolate the flaking surface and the discontinuous knapping pattern [94] represent the main shared features.

The emphasized variety of lamellar reduction strategies may be a result of the need to manufacture different end-products. Bladelets were used for multiple activities and some studies have proposed a correlation between size and function [66, 110, 141]. By comparison to the Early Aurignacian, Protoaurignacian bladelets are said to be large and straight [51, 55]. At Fumane, however, bladelets have varied dimensional and morphological attributes and large and rather straight blanks were found along with small and curved bladelets. The same variability has been shown to be characteristic of other industries, such as Mandrin [111], Isturitz [66], and Labeko Koba [56].

Blades represent the second goal of the Protoaurignacian lithic production system, and their frequency is always lower than that of bladelets. The flaked surface of blade cores was framed by at least one perpendicular flank; a feature that permitted the extraction of naturally backed blades and the use of neo-crests to shape the core convexities. Blades were extracted with direct marginal percussion and the striking platform usually remained flat. Faceted platforms, which are well-represented in Early Aurignacian assemblages of southwestern France [35, 142], are rare. Even if faceted platforms are not common outside of southwestern France [37, 51, 143, 144], the differences in the preparation of the core striking platform seem related to the production of more robust blades in Early Aurignacian assemblages [35, 36]. At Fumane, blades have variable morpho-metric attributes, but among retouched tools a selection of the bigger blanks, independent of their regularity and the presence of cortical remains, is verified. Among laterally-retouched blades, Aurignacian blades are present at variable degrees in most of the Protoaurignacian assemblages and are abundant at Abreda [114] and Tincova [116]. It does thus not seem to be a tool type restricted to Early Aurignacian assemblages, as is frequently argued [48, 145].

Flake production has been observed less often among Protoaurignacian industries and has generally received less attention in the available studies. At Fumane, most of the flakes recovered originated from the initialization and maintenance operations of blade and bladelet cores. For this reason, flake-tools were made mostly from by-products of the laminar reduction sequences, as demonstrated also at Siuren I [146]. At Arcy, an exclusive flake production has been described [49]. It was usually produced with low-quality raw material nodules or it could take place on exhausted laminar cores. At Morín, flakes were produced from discoid cores, and were used to manufacture side-scrapers and denticulates [147]. Generally, Protoaurignacian flake production appears to be marginal, as in most of the Early Aurignacian assemblages [35, 148].

### Testing models: Future research prospects

The Protoaurignacian is technologically consistent across its geographical extent. Bladelet production dictates the general organization of stone knapping, which is based on variable and, most cases, independent reduction strategies. The re-evaluation of the Protoaurignacian lithic technology has pointed out that this techno-complex shares a common technological background in the scope of lithic production with the Early Aurignacian and that no features are restricted to one of the two varieties. In the Early Aurignacian, bladelets are generally produced from carinated cores, even if the production could be carried out on prismatic and narrow-sided cores, as it is at Tuto-de-Camalhot [35], Barbas III (Ortega Cordellat, 2005), Les Cottés layer US 04 superior [122], Isturitz layers C4b1 and C4b2 [134, 149], Labeko Koba layer V [56], La Viña layer XIII [37], Geißenklösterle AHII [51], and Willendorf II AHIII [8, 19]. The higher frequency of carinated cores is probably a result of the need of different end-products. The major difference between the Protoaurignacian and Early Aurignacian appears to be more typological in nature, with retouched bladelets being less common in the Early Aurignacian.

Although the regional signatures of the Aurignacian techno-complex are far from being established, we argue that the clear-cut subdivision of two temporally consecutive technical traditions is unsustainable. The Swabian Aurignacian, for instance, has been associated with the Early Aurignacian of Aquitaine [51], although Hahn [150] has pointed out that the Aquitaine model does not apply to the region and Conard and Bolus [151] have emphasized the fact that the Aurignacian of the Swabian Jura is characterized by a strong local signature. In northern Italy, the development of the Protoaurignacian is still open to debate. At Mochi, preliminary results suggest that no clear cultural breaks are evident in the realm of the lithic assemblage between the two Aurignacian horizons [24]. Only antler exploitation and the manufacture of split-based bone points permit a differentiation between the upper and lower horizons [152]. Similar results have been reached in previous works at Fumane [67, 69]. The ongoing analyses on the upper (Proto)Aurignacian layers (D6 and D3) will be of primary importance in the understanding of the regional development of the Aurignacian in northeastern Italy.

In light of these observations and due to the narrow archaeological definition of Protoaurignacian and Early Aurignacian, the model proposed by Banks, d'Errico and Zilhao [39] is not applicable to all of Europe and should be viewed with caution. Future research will have to focus on the reasons for the quantitative differences found between Early Aurignacian and Protoaurignacian assemblages, by investigating the development of these techno-complexes on a regional perspective. Indeed, it is not clear whether all the industries described as Early Aurignacian are equivalent or if the earliest assemblages are comparable to the latest [25]. The cultural mosaic of lithic technologies at the beginning of the Upper Paleolithic could be explained in several ways. Among them, the progressive assimilation of the bladelet concept may have played a major role [55]. People’s high mobility may have permitted cultural interactions between different regional groups with exchanges of technological knowledge over large territories. In this regard, the association of the Aurignacian techno-complex with the spread of AMHs requires the design of a large-scale study that incorporates a detailed comparison of Eurasian Early Upper Paleolithic techno-complexes, such as the Baradostian [153–155], the Rostamian [155–157], and the Early Ahmarian [158].

## Conclusions

This extensive investigation of the lithic technology from the Protoaurignacian units A2-A1 at Fumane Cave and careful comparison with other assemblages confirms that the Protoaurignacian is a bladelet-dominated industry. Our study demonstrates that bladelet production is based on a broad range of reduction strategies that are not related to the reduction of larger blade cores, as postulated by Bon, Teyssandier and Bordes [48]. Blade and bladelet productions are, however, not strictly separated due to the presence of simultaneous reduction sequences, the recycling of some blade cores into bladelet cores, the selection of by-products of the bladelet production as blanks to manufacture common tools, and the production of a short sequence of blades on some initial bladelet cores prior to the main production phase. The Protoaurignacian appears to be technologically homogeneous, although regional signatures are noticeable in the typological variability of retouched bladelets [103] and in the importance given to certain platform reduction strategies, among which the preference towards the exploitation of the core longitudinal axis stands out.

In the light of recent radiocarbon dates, it is very likely that the Protoaurignacian and the Early Aurignacian coexisted for few millennia, probably in adjacent regions. This study suggests that no unique technological characteristics are restricted to either of the two techno-complexes. These results question the assumption that the Early Aurignacian evolved out of the Protoaurignacian [39]. Careful investigations carried out on a regional scale are the only way to clarify the relationships between human groups that inhabited Europe at the onset of the Upper Paleolithic. Being that the Protoaurignacian lithic assemblage of Fumane Cave has been extensively investigated and that its technological spectrum encompasses all of the variability that has been verified in all Protoaurignacian assemblages, it should be used as a reference site for the identification of inter-regional variability and for large-scale comparisons among contemporaneous Eurasian techno-complexes.

## Supporting information

S1 File List of all lithic artifacts analyzed in this paper. For each artifact is given A. Falcucci’s database number, basic dataclass, technological classification, cortex coverage, breakage class, and all individual measurements. Measurements include: length (only for complete artifacts), width, and thickness of blanks and tools, and width and thickness of preserved platforms.
(XLSX)

S1 Fig Plan view of the cave. Squares colored yellow are square meters where all cores, all tools and tool fragments, all complete and almost complete blades and bladelets, and all by-products deemed to have had a significant role in the reduction process were studied. Additionally, in squares colored brown all blades and bladelets greater than 1.5 cm regardless of the fragmentation index and all flakes with preserved butts greater than 2.0 cm were analyzed.
(PDF)

S2 Fig Core diacritic analyses. Schematic drawings of semi-circumferential blade (a) and bladelet (b, e) cores, wide-faced flat blade-bladelet (c) and blade (h) cores, narrow-sided bladelet cores (d, i), transverse carinated bladelet core (f), and multi-platform bladelet core (g). See individual captions for interpretation of core reduction procedures and the legend for explanation of the symbols and graphic criteria used to draw cores (drawings: A. Falcucci).
(PDF)

S3 Fig Comparison between the distribution of blade thickness values (in millimeters; blue) and bladelet thickness values (in millimeters; green). (PDF)

S1 Table Summary of length measurements across complete blanks (flakes, blades, and bladelets together) with different grades of cortex coverage. SE: standard error; SD: standard deviation.
(PDF)

S2 Table Summary of metric attributes of blades made from Oolithic flint and blades made from all other raw material types. SE: standard error; SD: standard deviation.
(PDF)

S3 Table Summary of length measurements across complete technical flakes with blade, bladelet, and simultaneous blade-bladelet scars. Complete technical flakes with undetermined scars (n = 6) are excluded. SE: standard error; SD: standard deviation.
(PDF)

## References

[1] Mellars P. Archeology and the dispersal of modern humans in Europe: Deconstructing the "Aurignacian". Evol Anthropol. 2006;15(5):167–82. doi: 10.1002/evan.20103 PubMed PMID: WOS:000241915700004.

[2] Davies W. Re-evaluating the Aurignacian as an expression of modern human mobility and dispersal In: Mellars P, Boyle K, Bar-Yosef O, Stringer C, editors. Rethinking the Human Revolution: New Behavioural and Biological Perspectives on the Origin and Dispersal of Modern Humans. Cambridge, UK: McDonald Institute for Archaeological Research, University of Cambridge; 2007 p. 263–74.

[3] Hublin J-J. The modern human colonization of western Eurasia: when and where? Quaternary Sci Rev. 2015;118:194–210. doi: 10.1016/j.quascirev.2014.08.011

[4] Conard N. The Timing of Cultural Innovations and the Dispersal of Modern Humans in Europe. In: Brauer A, Negendank JFW, Bohm M, editors. Proceedings of the DEUQUA-Meeting August 26–28 2002 Potsdam, Germany. 6. Oxford: Terra Nova; 2002. p. 82–94.

[5] Hoffecker JF. The spread of modern humans in Europe. Proceedings of the National Academy of Sciences. 2009;106(38):16040–5. doi: 10.1073/pnas.0903446106 19571003 10.1073/pnas.0903446106 PMC2752585

[6] Tsanova T, Zwyns N, Eizenberg L, Teyssandier N, Le Brun-Ricalens F, Otte M. Le plus petit dénominateur commun : réflexion sur la variabilité des ensembles lamellaires du Paléolithique supérieur ancien d’Eurasie. Un bilan autour des exemples de Kozarnika (Est des Balkans) et Yafteh (Zagros central). L'Anthropologie. 2012;116(4):469–509. doi: 10.1016/j.anthro.2011.10.005

[7] Kadowaki S, Omori T, Nishiaki Y. Variability in Early Ahmarian lithic technology and its implications for the model of a Levantine origin of the Protoaurignacian. J Hum Evol. 2015;82:67–87. doi: 10.1016/j.jhevol.2015.02.017 . 25924809 10.1016/j.jhevol.2015.02.017

[8] Nigst PR. The Early Upper Palaeolithic of the Middle Danube Region. Leiden: Leiden University Press; 2012.

[9] Bar-Yosef O. Neanderthals and modern humans: A different interpretation In: Conard N, editor. When Neanderthals and Modern Humans Met. Tübingen, Germany: Kerns Verlag; 2012 p. 467–82.

[10] Tostevin G. Seeing Lithics: A Middle-Range Theory for Testing for Cultural Transmission in the Pleistocene. Oxford, UK: Oxbow Books; 2013.

[11] Skrdla P. Comparison of Boker Tachtit and Stranska skala MP/UP transitional industries. Journal of the Israel Prehistoric Society. 2003;33:37–73.

[12] Benazzi S, Douka K, Fornai C, Bauer CC, Kullmer O, Svoboda J, et al Early dispersal of modern humans in Europe and implications for Neanderthal behaviour. Nature. 2011;479(7374):525–8. doi: 10.1038/nature10617 . 22048311 10.1038/nature10617

[13] Zilhão J, Banks WE, d’Errico F, Gioia P. Analysis of Site Formation and Assemblage Integrity Does Not Support Attribution of the Uluzzian to Modern Humans at Grotta del Cavallo. PLOS ONE. 2015;10(7):e0131181 doi: 10.1371/journal.pone.0131181 26154139 10.1371/journal.pone.0131181 PMC4495988

[14] Benazzi S, Bailey SE, Peresani M, Mannino MA, Romandini M, Richards MP, et al Middle Paleolithic and Uluzzian human remains from Fumane Cave, Italy. J Hum Evol. 2014;70:61–8. http://doi.org/10.1016/j.jhevol.2014.03.001. 24666601 10.1016/j.jhevol.2014.03.001

[15] Peresani M, Cristiani E, Romandini M. The Uluzzian technology of Grotta di Fumane and its implication for reconstructing cultural dynamics in the Middle-Upper Palaeolithic transition of Western Eurasia. J Hum Evol. 2016;91:36–56. doi: 10.1016/j.jhevol.2015.10.012 . 26852812 10.1016/j.jhevol.2015.10.012

[16] Bailey SE, Weaver TD, Hublin JJ. Who made the Aurignacian and other early Upper Paleolithic industries? J Hum Evol. 2009;57(1):11–26. doi: 10.1016/j.jhevol.2009.02.003 PubMed PMID: WOS:000268652600002. 19476971 10.1016/j.jhevol.2009.02.003

[17] Benazzi S, Slon V, Talamo S, Negrino F, Peresani M, Bailey SE, et al The makers of the Protoaurignacian and implications for Neandertal extinction. Science (New York, NY). 2015;348(6236):793–6. Epub 2015/04/25. doi: 10.1126/science.aaa2773 . 25908660 10.1126/science.aaa2773

[18] Higham T, Basell L, Jacobi R, Wood R, Ramsey CB, Conard NJ. Testing models for the beginnings of the Aurignacian and the advent of figurative art and music: The radiocarbon chronology of Geissenklosterle. J Hum Evol. 2012;62(6):664–76. doi: 10.1016/j.jhevol.2012.03.003 PubMed PMID: WOS:000306046700002. 22575323 10.1016/j.jhevol.2012.03.003

[19] Nigst PR, Haesaerts P, Damblon F, Frank-Fellner C, Mallol C, Viola B, et al Early modern human settlement of Europe north of the Alps occurred 43,500 years ago in a cold steppe-type environment. Proceedings of the National Academy of Sciences. 2014;111(40):14394–9. doi: 10.1073/pnas.1412201111 25246543 10.1073/pnas.1412201111 PMC4209988

[20] Conard NJ, Bolus M. Radiocarbon dating the appearance of modern humans and timing of cultural innovations in Europe: new results and new challenges. J Hum Evol. 2003;44(3):331–71. . 12657520 10.1016/s0047-2484(02)00202-6

[21] Davies W, White D, Lewis M, Stringer C. Evaluating the transitional mosaic: frameworks of change from Neanderthals to Homo sapiens in eastern Europe. Quaternary Sci Rev. 2015;118(Supplement C):211–42. https://doi.org/10.1016/j.quascirev.2014.12.003.

[22] Davies W, Hedges RE. Dating a type site: Fitting Szeleta Cave into its regional chronometric context. Praehistoria 2008;9–10:35–45.

[23] Szmidt CC, Normand C, Burr GS, Hodgins GWL, LaMotta S. AMS C-14 dating the Protoaurignacian/Early Aurignacian of Isturitz, France. Implications for Neanderthal-modern human interaction and the timing of technical and cultural innovations in Europe. J Archaeol Sci. 2010;37(4):758–68. doi: 10.1016/j.jas.2009.11.006 PubMed PMID: WOS:000276113400010.

[24] Douka K, Grimaldi S, Boschian G, del Lucchese A, Higham TF. A new chronostratigraphic framework for the Upper Palaeolithic of Riparo Mochi (Italy). J Hum Evol. 2012;62(2):286–99. doi: 10.1016/j.jhevol.2011.11.009 . 22189428 10.1016/j.jhevol.2011.11.009

[25] Wood RE, Arrizabalaga A, Camps M, Fallon S, Iriarte-Chiapusso MJ, Jones R, et al The chronology of the earliest Upper Palaeolithic in northern Iberia: New insights from L'Arbreda, Labeko Koba and La Vina. J Hum Evol. 2014;69:91–109. doi: 10.1016/j.jhevol.2013.12.017 . 24636733 10.1016/j.jhevol.2013.12.017

[26] Higham T, Douka K, Wood R, Ramsey CB, Brock F, Basell L, et al The timing and spatiotemporal patterning of Neanderthal disappearance. Nature. 2014;512(7514):306–9. doi: 10.1038/nature13621 . 25143113 10.1038/nature13621

[27] Douka K, Higham TF, Wood R, Boscato P, Gambassini P, Karkanas P, et al On the chronology of the Uluzzian. J Hum Evol. 2014;68:1–13. doi: 10.1016/j.jhevol.2013.12.007 . 24513033 10.1016/j.jhevol.2013.12.007

[28] Breuil H, editor Les subdivisions du Paléolithique Supérieur et leur signification. Congrès International d’Anthropologie et d’Archéologie Préhistorique (XIVe session); 1912.

[29] Peyrony D. Les industries ‘aurignaciennes’ dans le bassin de la Vézère. Bulletin de la Société Préhistorique Française. 1933;30:543–59.

[30] Garrod D. The Upper Palaeolithic in the light of recent discovery. Proceedings of the Prehistoric Society. 1938;4:1–26.

[31] Sonneville-Bordes de D. Le Paléolithique supérieur en Périgord. Bordeaux, France: Delmas,; 1960.

[32] Delporte H. L’Abri du Facteur à Tursac (Dordogne) I: étude générale. Gallia Préhistoire. 1968;11:1–112.

[33] Laplace G. Recherches Sur l’origine et l’évolution des complexes leptolithiques. Paris, France: De Bocard; 1966.

[34] Bon F. Les termes de l’Aurignacien In: Bon F, Maillo Fernandez JM, Ortega-Cobos D, editors. Autour des concepts de Protoaurignacien, d’Aurignacien archaïque, initial et ancien Unité et variabilité des comportements techniques des premiers groupes d’hommes modernes dans le sud de la France et le nord de l’Espagne. Madrid, Spain: UNED; 2006 p. 39–65.

[35] Bon F. L'Aurignacien entre mer et océan: réflexion sur l'unité des phases anciennes de l'Aurignacien dans le Sud de la France. Paris, France: Société préhistorique française; 2002.

[36] Bordes JG. News from the West: a reevaluation of the classical Aurignacian sequence of the Périgord In: Bar-Yosef O, Zilhão J, editors. Towards a Definition of the Aurignacian. 45 Lisbon, Portugal: IPA; 2006 p. 147–71.

[37] Santamaría D. La transición del Paleolítico medio al superior en Asturias El Abrigo de La Viña (La Manzaneda, Oviedo) y la Cueva de El Sidrón (Borines, Piloña). Servicio de Publicaciones de la Universidad de Oviedo Oviedo; 2012.

[38] Arrizabalaga A, Altuna J. Labeko Koba (País Vasco). Hienas y humanos en los albores del Paleolítico superior. Soc. de Ciencias Aranzadi; 2000.

[39] Banks WE, d'Errico F, Zilhao J. Human-climate interaction during the Early Upper Palaeolithic: Testing the hypothesis of an adaptive shift between the Proto-Aurignacian and the Early Aurignacian. J Hum Evol. 2013;64(3):232–. doi: 10.1016/j.jhevol.2013.01.001 PubMed PMID: WOS:000316437900006. 10.1016/j.jhevol.2012.10.001 23245623

[40] Higham T, Wood R, Moreau L, Conard N, Ramsey CB. Comments on 'Human climate interaction during the early Upper Paleolithic: Testing the hypothesis of an adaptive shift between the Proto-Aurignacian and the Early Aurignacian' by Banks et al. J Hum Evol. 2013;65(6):806–9. doi: 10.1016/j.jhevol.2013.06.010 PubMed PMID: WOS:000328721100010. 24120081 10.1016/j.jhevol.2013.06.010

[41] Ronchitelli A, Benazzi S, Boscato P, Douka K, Moroni A. Comments on "Human-climate interaction during the Early Upper Paleolithic: Testing the hypothesis of an adaptive shift between the Proto-Aurignacian and the Early Aurignacian" by William E. Banks, Francesco d'Errico, Joao Zilhao. J Hum Evol. 2014;73:107–11. doi: 10.1016/j.jhevol.2013.12.010 PubMed PMID: WOS:000340985100012. 24529865 10.1016/j.jhevol.2013.12.010

[42] Mellars P. Neanderthals and the modern human colonization of Europe. Nature. 2004;432(7016):461–5. doi: 10.1038/nature03103 15565144 10.1038/nature03103

[43] Bertola S, Broglio A, Cristiani E, de Stefani M, Gurioli F, Negrino F, et al La diffusione del primo Aurignaziano a sud dell'arco alpino. Preistoria Alpina. 2013;47:17–30.

[44] Mellars P. A new radiocarbon revolution and the dispersal of modern humans in Eurasia. Nature. 2006;439(7079):931–5. doi: 10.1038/nature04521 16495989 10.1038/nature04521

[45] Conard N, Bolus M. Chronicling modern human’s arrival in Europe. Science (New York, NY). 2015 doi: 10.1126/science.aab0234 25908661 10.1126/science.aab0234

[46] Le Brun-Ricalens F. Productions lamellaires attribuées à l’Aurignacien. Luxembourg: MNHA; 2005.

[47] Bordes J-G. Les interstratifications Châtelperronien/Aurignacien du Roc-de-Combe et du Piage (Lot, France): analyse taphonomique des industries lithiques, implications archéologiques 2002.

[48] Bon F, Teyssandier N, Bordes JG. La signification culturelle des équipements lithiques In: Otte M, editor. Les Aurignaciens. Paris, France: Errance; 2010 p. 46–65.

[49] Bon F, Bodu P. Analyse technologique du débitage aurignacien In: Schmider B, editor. L’Aurignacien de la grotte du Renne Les fouilles d’André Leroi-Gourhan à Arcy-sur-Cure (Yonne). Paris, France: CNRS; 2002 p. 115–33.

[50] Demars PY, Laurent P. Types d’outils lithiques du Paléolithique supérieur en Europe. Paris, France: CNRS; 1992.

[51] Teyssandier N. En route vers l'Ouest Les débuts de l'Aurignacien en Europe. John and Erica Hedges Ltd.; 2007.

[52] Teyssandier N. Revolution or evolution: the emergence of the Upper Paleolithic in Europe. World Archaeol. 2008;40(4):493–519. doi: 10.1080/00438240802452676

[53] Le Brun-Ricalens F. Chronique d'une reconnaissance attendue. Outils "carénés", outils "nucléiformes": nucléus à lamelles. Bilan après un siècle de recherches typologiques, technologiques et tracéologiques In: Le Brun-Ricalens F, editor. Productions lamellaires attribuées à l’Aurignacien. Luxembourg: MNHA; 2005.

[54] Bon F. Little big tool. Enquete autour du succés de la lamelle In: Le Brun-Ricalens F, editor. Productions lamellaires attribuées à l’Aurignacien. Luxembourg: MNHA; 2005 p. 479–84.

[55] Le Brun-Ricalens F, Bordes JG, Eizenberg L. A crossed-glance between southern European and Middle-Near Eastern early Upper Palaeolithic lithic technocomplexes. Existing models, new perspectives In: Camps M, Szmidt C, editors. The Mediterranean from fifty thousand to twenty-five thousand BP: Turning points and new directions. Oxford: Oxbow Books; 2009 p. 11–33.

[56] Tafelmaier I. Technological variability at the beginning of the Aurignacian in Northern Spain: Wissenschaftliche Schriften des Neanderthal Museums; 2017.

[57] Moreau L, Odar B, Higham T, Horvat A, Pirkmajer D, Turk P. Reassessing the Aurignacian of Slovenia: Techno-economic behaviour and direct dating of osseous projectile points. J Hum Evol. 2015;78:158–80. doi: 10.1016/j.jhevol.2014.09.007 PubMed PMID: WOS:000349063600013. 25498105 10.1016/j.jhevol.2014.09.007

[58] Szmidt CC, Brou L, Jaccottey L. Direct radiocarbon (AMS) dating of split-based points from the (Proto)Aurignacian of Trou de la Mere Clochette, Northeastern France. Implications for the characterization of the Aurignacian and the timing of technical innovations in Europe. J Archaeol Sci. 2010;37(12):3320–37. doi: 10.1016/j.jas.2010.08.001 PubMed PMID: WOS:000283903500035.

[59] Maroto J, Soler N, Fullola JM. Cultural Change Between Middle and Upper Palaeolithic in Catalonia In: Carbonell E, Vaquero M, editors. The last neandertals, the first anatomically modern humans: A tale about the diversity Cultural change and humans evolution: the crisis at 40 KA BP. Tarragona, Spain: Universitat Rovira i Virgili; 1996 p. 219–50.

[60] Conard NJ. A female figurine from the basal Aurignacian of Hohle Fels Cave in southwestern Germany. Nature. 2009;459(7244):248–52. doi: 10.1038/nature07995 PubMed PMID: WOS:000266036100040. 19444215 10.1038/nature07995

[61] Kuhn SL, Stiner MC. The earliest Aurignacian of Riparo Mochi (Liguria, Italy). Curr Anthropol. 1998;39:S175–S89. doi: 10.1086/204694 PubMed PMID: WOS:000073731300007.

[62] Zilhão J. The Emergence of Ornaments and Art: An Archaeological Perspective on the Origins of “Behavioral Modernity”. Journal of Archaeological Research. 2007;15(1):1–54. doi: 10.1007/s10814-006-9008-1

[63] Vanhaeren M, d'Errico F. Aurignacian ethno-linguistic geography of Europe revealed by personal ornaments. J Archaeol Sci. 2006;33(8):1105–28. doi: 10.1016/j.jas.2005.11.017 PubMed PMID: WOS:000238294900008.

[64] Demidenko YE, Otte M, Noiret P. Siuren I Rock-Shelter. From Late Middle Paleolithic and Early Upper Paleolithic to Epi-Paleolithic in Crimea. Liège, Belgium: ERAUL; 2012.

[65] Sitlivy V, Chabai V, Anghelinu M, Uthmeier T, Kels H, Hilgers A, et al The earliest Aurignacian in Romania: New investigations at the open air site of Româneşti-Dumbrăviţa I (Banat). Quartär. 2012;59:85–130. doi: 10.7485/QU59_4

[66] Normand C, O’Farrell M, Rios Garaizar J. Quelle(s) utilisation(s) pour les productions lamellaires de l’Aurignacien Archaique? Quelques données et réflexions à partir des exemplaires de la grotte d’Isturiz (Pyrénées-Atlantiques; France). Palethnologie. 2008;1(Recherches Sur les armatures de projectiles du Paléolithique supérieur au Néolithique):7–46.

[67] Broglio A, Bertola S, de Stefani M, Marini D, Lemorini C, Rossetti P. La production lamellaire et les armatures lamellaires de l’Aurignacien ancien de la grotte de Fumane (Monts Lessini, Vénétie) In: Le Brun-Ricalens F, editor. Productions lamellaires attribuées à l’Aurignacien. 415–436. Luxembourg: MNHA; 2005.

[68] Broglio A, Dalmeri G. Pitture paleolitiche nelle Prealpi Venete: Grotta di Fumane e Riparo Dalmeri. Verona, Italy: Mem. Verona, Sezione Scienze dell'Uomo; 2005.

[69] Higham T, Brock F, Peresani M, Broglio A, Wood R, Douka K. Problems with radiocarbon dating the Middle to Upper Palaeolithic transition in Italy. Quaternary Sci Rev. 2009;28(13–14):1257–67. http://doi.org/10.1016/j.quascirev.2008.12.018.

[70] López-García JM, dalla Valle C, Cremaschi M, Peresani M. Reconstruction of the Neanderthal and Modern Human landscape and climate from the Fumane cave sequence (Verona, Italy) using small-mammal assemblages. Quaternary Sci Rev. 2015;128:1–13. http://doi.org/10.1016/j.quascirev.2015.09.013.

[71] Peresani M. Fifty thousand years of flint knapping and tool shaping across the Mousterian and Uluzzian sequence of Fumane cave. Quatern Int. 2012;247:125–50. http://doi.org/10.1016/j.quaint.2011.02.006.

[72] Broglio A, Cremaschi M, Peresani M, Bertola S, Bolognesi L, De Stefani M, et al. L’Aurignacien dans le territoire préalpin: la Grotte de Fumane. In: Vasilev SA, Soffer O, Kozlowski JK, editors. Perceived landscapes and built environments The cultural geography of Late Palaeolithic Eurasia. 1122: British Archaeological Reports, International Series; 2003. p. 93–104.

[73] Peresani M, Centi E, Di Taranto L. Blades, bladelets and flakes: A case of variability in tool design at the dawn of the Middle–Upper Palaeolithic transition in Italy. Cr Palevol. 2013;12(4):211–21. http://doi.org/10.1016/j.crpv.2013.02.005.

[74] Peresani M. A New Cultural Frontier for the Last Neanderthals: The Uluzzian in Northern Italy. Curr Anthropol. 2008;49(4):725–31. doi: 10.1086/588540

[75] Cavallo G, Fontana F, Gonzato F, Peresani M, Riccardi MP, Zorzin R. Textural, microstructural and compositional characteristics of Fe-based geomaterials and Upper Palaeolithic ocher in the Lessini Mountains, Northeast Italy: implications for provenance studies. Geoarchaeology. 2017;32(4):437–55. doi: 10.1002/gea.21617

[76] Cavallo G, Fontana F, Gialanella S, Gonzato F, Riccardi MP, Zorzin R, et al Heat treatment of mineral pigment during Upper Palaeolithic in North-Eastern Italy. Archaeometry. Accepted manuscript. doi: 10.1111/arcm.12058

[77] Broglio A, De Stefani M, Gurioli F, Pallecchi P, Giachi G, Higham T, et al The decoration of Fumane Cave in the picture of the Aurignacian art. Anthropologie. 2009;113(5):753–61. doi: 10.1016/j.anthro.2009.09.016 PubMed PMID: WOS:000273288700003.

[78] Broglio A, Bertola S, de Stefani M, Gurioli F. Le strutture d’abitato aurignaziane della Grotta di Fumane. Dialektikê, Cahiers de Typologie analytique. 2006;(Servei d’Investigacions Arquerlògiques i Prehistòriques):27–43.

[79] Broglio A, de Stefani M, Tagliacozzo A, Gurioli F, Facciolo A. Aurignacian dwelling structures, hunting strategies and seasonality in the Fumane Cave (Lessini Mountains) In: Vasil'ev SA, Popov VV, Anikovich MV, Praslov ND, Sinitsyn AA, Hoffecker JF, editors. Kostenki and the Early Upper Paleolithic of Eurasia: General Trends, Local Developments. Saint Petersburg: Nestor-Historia; 2006 p. 263–8.

[80] Gurioli F, Cilli C, Giacobini G, Broglio A. Le conchiglie perforate aurignaziane della Grotta di Fumane (VR). IV Convegno Nazionale di Archeozoologia; Pordenone, Italy2005. p. 59–65.

[81] Bartolomei G, Broglio A, Cassoli P, Cremaschi M, Giacobini G, Malerba G, et al Risultati preliminari delle nuove ricerche al Riparo di Fumane. Annuario storico della Valpolicella. 1992:9–64.

[82] Fiore I, Gala M, Tagliacozzo A. Ecology and subsistence strategies in the eastern Italian alps during the middle Palaeolithic. International Journal of Osteoarchaeology. 2004;14:273–86.

[83] Bertola S. Contributo allo studio del comportamento dei primi gruppi di Homo sapiens sapiens diffusi in Europa. Sfruttamento della selce, produzione dei supporti lamellari, confezione delle armature litiche nel sito aurignaziano della Grotta di Fumane nei Monti Lessini (Verona): University of Bologna; 2001.

[84] Boëda E, Geneste J-M, Meignen L. Identification de chaînes opératoires lithiques du Paléolithique ancien et moyen. Paléo. 1990;2(1):43–80. doi: 10.3406/pal.1990.988

[85] Inizan ML, Reduron M, Roche H, Tixier J. Technologie de la pierre taillée Préhistoire de la pierre taillée. Meudon: CREP; 1995.

[86] Shott MJ. Chaîne opératoire and reduction sequence. Lithic Technology. 2003;28:95–106.

[87] Soressi M, Geneste J-M. The history and efficacy of the chaîne opératoire approach to lithic analysis: Studying techniques to reveal past societies in an evolutionary perspective. PaleoAnthropology. 2011:334–50.

[88] Conard NJ, Adler DS. Lithic Reduction and Hominid Behavior in the Middle Paleolithic of the Rhineland. J Anthropol Res. 1997;53(2):147–75. doi: 10.1086/jar.53.2.3631275

[89] Andrefsky W. Lithics: Macroscopic approaches to analysis. Cambridge: Cambridge University; 1998.

[90] Odell GH. Lithic analysis. New York: Springer; 2004.

[91] Zwyns N. Laminar technology and the onset of the Upper Paleolithic in the Altai, Siberia. Leiden: Leiden University Press; 2012.

[92] Dauvois M. Précis de dessin dynamique et structural des industries lithiques préhistoriques. Périgueux: Pierre Fanlac; 1976.

[93] Roussel M. Normes et variations de la production lithique durant le Chatelperronien: la séquence de la Grande-Roche-de-la-Plématrie à Quinçay (Vienne) University of Paris Ouest-Nanterre; 2011.

[94] Falcucci A, Peresani M. Protoaurignacian core reduction procedures: blade and bladelet technologies at Fumane Cave. submitted.

[95] Pelegrin J. Les techniques de débitage laminaire au Tardiglaciaire: critères de diagnose et quelques réflexions. In: Valentin B, Bodu P, Christensen M, editors. L’Europe centrale et septentrionale au Tardiglaciaire Confrontation des modèles régionaux. Nemours: Mémoires du musée de Préhistoire d’Île-de-France; 2000. p. 73–86.

[96] Soriano S, Villa P, Wadley L. Blade technology and tool forms in the Middle Stone Age of South Africa: the Howiesons Poort and post-Howiesons Poort at Rose Cottage Cave. J Archaeol Sci. 2007;34(5):681–703. doi: 10.1016/j.jas.2006.06.017

[97] Conard NJ, Soressi M, Parkington JE, Wurz S, Yates R. A unified lithic taxonomy based on patterns of core reduction. South African Archaeological Bulletin South African Archaeological Bulletin. 2004;59:12–6.

[98] Tixier J. Typologie de l’Epipaléolithique du Maghreb. Paris: Mémoires du Centre de Recherches anthropologiques et préhistoriques et ethnographiques; 1963.

[99] Holm S. A simple sequentially rejective multiple test procedure. Scandinavian Journal of Statistics. 1979;6:65–70.

[100] Weissmüller W. Die Silexartefakte der Unteren Schichten der Sesselfelsgrotte Ein Beitrag zum Problem des Moustérien. Saarbrücken: Saarbrücker Druckerei und Verlag (Quartär-Bibliothek 6); 1995.

[101] Walczak J. La question des styles techniques durant le Mésolithique: remarques générales sur le style tardenoisien de Coincy et sur sa "valeur humaine ". Bulletin de la Société préhistorique française. 1998:203–20.

[102] Porraz G, Igreja M, Schmidt P, E. PJ. A shape to the microlithic Robberg from Elands Bay Cave (South Africa). Southern African Humanities. 2016;29:203–47.

[103] Falcucci A, Peresani M, Roussel M, Normand C, Soressi M. What’s the point? Retouched bladelet variability in the Protoaurignacian. Results from Fumane, Isturitz, and Les Cottés. Archaeological and Anthropological Sciences. 2016 doi: 10.1007/s12520-016-0365-5

[104] Pelegrin J. Technologie lithique: le Chatelperronien de Roc-de-Combe (lot) et de La Cote (Dordogne). Paris: CNRS Editions; 1995.

[105] Roussel M, Bourguignon L, Soressi M. Identification par l’expérimentation de la percussion au percuteur de calcaire au Paléolithique moyen: le cas du façonnage des racloirs bifaciaux Quina de Chez Pinaud (Jonzac, Charente-Maritime). Bulletin de la Société préhistorique française. 2009:219–38.

[106] Driscoll K, García-Rojas M. Their lips are sealed: identifying hard stone, soft stone, and antler hammer direct percussion in Palaeolithic prismatic blade production. J Archaeol Sci. 2014;47:134–41. doi: 10.1016/j.jas.2014.04.008

[107] Magnani M, Rezek Z, Lin SC, Chan A, Dibble HL. Flake variation in relation to the application of force. J Archaeol Sci. 2014;46(Supplement C):37–49. https://doi.org/10.1016/j.jas.2014.02.029.

[108] Dini M, Baills H, Conforti J, Tozzi C. Le Protoaurignacien de la Grotte La Fabbrica (Grosseto, Italie) dans le contexte de l’arc nord méditerranéen. L'Anthropologie. 2012;116(4):550–74. doi: 10.1016/j.anthro.2012.10.003

[109] Gambassini P. Il Paleolitico di Castelcivita: Culture e Ambiente. Naples: Electa; 1997.

[110] Porraz G, Simon P, Pasquini A. Identité technique et comportements économiques des groupes proto-aurignaciens à la grotte de l’Observatoire (principauté de Monaco). Gallia préhistoire. 2010:33–59.

[111] Slimak L, Pesesse D, Giraud Y. La grotte Mandrin et les premières occupations du Paléolithique supérieur en Occitanie orientale In: Bon F, Maillo Fernandez JM, Ortega Cobos D, editors. Autour des concepts de Proto-Aurignacien, d’Aurignacien initial et ancien: unité et variabilité des comportements techniques des premiers groupes d’hommes modernes dans le sud de la France et le nord de l’Espagne,. Madrid: UNED; 2006 p. 237–59.

[112] Slimak L, Pesesse D, Giraud Y. Reconnaissance d'une installation du Protoaurignacien en vallée du Rhône. Implications sur nos connaissances concernant les premiers hommes modernes en France méditerranéenne. Cr Palevol. 2006;5(7):909–17. http://doi.org/10.1016/j.crpv.2006.05.002.

[113] Sicard S. L’Aurignacien archaïque de l’Esquicho-Grapaou: analyse typo-technologique du débitage [Master dissertation]: Université de Paris I—Panthéon-Sorbonne; 1994.

[114] Ortega Cobos D, Soler N, Maroto J. La production de lamelles pendant l’Aurignacien archaïque dans la grotte de l’Arbreda: organisation de la production, variabilité des méthodes et des objectifs In: Le Brun-Ricalens F, editor. Productions lamellaires attribuées à l’Aurignacien. Luxembourg: MNHA; 2005 p. 359–73.

[115] Paris C. La production de lamelles Dufour dans la couche VII d’Arcy-sur-cure (Yonne, aurignacien): Université de Paris I—Panthéon-Sorbonne; 2005.

[116] Sitlivy V, Anghelinu M, Chabai V, Nitjă L, Uthmeier T, Hauck T, et al Placing the Aurignacian from Banat (Soutwestern Romania) into the European Early Upper Paleolithic context In: Otte M, Le Brun-Ricalens F, editors. Modes de contacts et de déplacements au Paléolithique eurasiatique. 140 Liège: ERAUL; 2014 p. 243–77.

[117] Zwyns N. Small laminar blanks at Siuren I rockshelter: technological and comparative approach In: Demidenko YE, Otte M, Noiret P, editors. Siuren I rock-shelter. 129 Liège: ERAUL; 2012 p. 359–73.

[118] Bataille G. Extracting the “Proto” from the Aurignacian. Distinct Production Sequences of Blades and Bladelets in the Lower Aurignacian Phase of Siuren I, Units H and G (Crimea). Mitteilungen der Gesellschaft für Urgeschichte. 2017;25:49–85.

[119] Maillo-Fernandez JM. La production lamellaire de l’Aurignacien de la grotte Morín (Cantabrie, Espagne) In: Le Brun-Ricalens F, editor. Productions lamellaires attribuées à l’Aurignacien: chaînes opératoires et perspectives technoculturelles. Luxembourg: MNHA; 2005 p. 339–57.

[120] Tsanova T. Les débuts du Paléolithique supérieur dans l’Est des Balkans. Réflexion à partir de l’étude taphonomique et technoéconomique des ensembles lithiques de Bacho Kiro (couche 11), Temnata (couches VI et 4) et Kozarnika (niveau VII). Oxford: BAR International Series 2008.

[121] Bordes JG. La séquence aurignacienne du Nord de l’Aquitaine: variabilité des productions lamellaires à Caminade-Est, roc-de- Combe, Le Piage et Corbiac-Vignoble II In: Le Brun-Ricalens F, editor. Productions lamellaires attribuées à l’Aurignacien: chaînes opératoires et perspectives technoculturelles. Luxembourg: MNHA; 2005 p. 123–54.

[122] Roussel M, Soressi M. Une nouvelle séquence du Paléolithique supérieur ancien aux marges sud-ouest du Bassin parisien: Les Cottés dans la Vienne. In: Bodu P, Chehmana L, L. K, Mevel L, Soriano S, Teyssandier N, editors. Le Paléolithique supérieur ancien de l’Europe du Nord-Ouest: Mémoire LVI de la Société préhistorique française; 2013. p. 283–97.

[123] Chadelle J-P. Productions "intriquées" de lames et lamelles dans l'Aurignacien de Champ-Parel Locus 3 (Bergerac, Dordogne) In: Le Brun-Ricalens F, editor. Productions lamellaires attribuées à l’Aurignacien. Luxembourg: MNHA; 2005 p. 193–208.

[124] Le Brun-Ricalens F. L'occupation aurignacienne d'Hui (Beauville, Lot-et-Garonne). Bulletin de la Société préhistorique française. 1990;87(9):275–82. doi: 10.3406/bspf.1990.9448

[125] Le Brun-Ricalens F. Reconnaissance d'un "concept technoculturel" de l'Aurignacien ancien? Modalités, unités et variabilités des productions lamellaires du site d'Hui (Beauville, Lot-et-Garonne, France): significations et implications In: Le Brun-Ricalens F, editor. Productions lamellaires attribuées à l’Aurignacien. Luxembourg: MNHA; 2005 p. 157–90.

[126] Perpère M, Schmider B. L´outillage litique In: Schmider B, editor. L’Aurignacien de la grotte du Renne Les fouilles d’André Leroi-Gourhan à Arcy-sur-Cure (Yonne). 34 Paris: CNRS Éditions, Gallia préhistoire; 2002 p. 143–95.

[127] Anderson L, Bon F, Bordes JG, pasquini A, Slimak L, Teyssandier N. Relier des espaces, construire de nouveaux réseaux: aux origines du Protoaurignacien et des débuts du Paléolithique supérieur en Europe occidentale In: Naudinot N, Meignen L, Binder G, Querré G, editors. Les systèmes de mobilité de la Préhistoire au Moyen Âge XXXVe rencontres internationales d’archéologie et d’histoire d’Antibes. Antibes: Éditions APDCA; 2015.

[128] Riel-Salvatore J, Negrino F. Early Upper Paleolithic population dynamics and raw material procument patterns in Italiy In: Camps M, Szmidt C, editors. The Mediterranean from 50000 to 25000 BP—Turning points and new directions. Oxford: Oxbow Books; 2009 p. 211–30.

[129] Sicard S. La Louza (Gard): approche techno-fonctionnelle d'une chaine operatoire aurignacienne [D.E.A. dissertation]: Université de Paris I—Panthéon-Sorbonne; 1995.

[130] Bietti A, Negrino F. L’Aurignacien et le Gravettien du Riparo Mochi, l’Aurignacien du Riparo Bombrini: comparaisons et nouvelles perspectives. Arch Inst Paléontologie Humaine. 2008;39:133–40.

[131] Grimaldi S, Porraz G, Santaniello F. Raw material procurement and land use in the northern Mediterranean Arc: insight from the first Proto-Aurignacian of Riparo Mochi (Balzi Rossi, Italy). Quartär. 2014;61:113–27. doi: 10.7485/QU61_06

[132] Bazile F. La composante lamellaire dans l'Aurignacien Initial de la France méditerranéenne In: Le Brun-Ricalens F, editor. Productions lamellaires attribuées à l’Aurignacien. Luxembourg: MNHA; 2005 p. 325–36.

[133] Maillo-Fernandez JM. Archaic Aurignacian lithic technology in Cueva Morín (Cantabria, Spain) In: Bar-Yosef O, Zilhão J, editors. Towards a Definition of the Aurignacian. Lisbon, Portugal: IPA; 2006 p. 109–28.

[134] Normand C, Turq A. L’Aurignacien de la grotte d’Isturitz (France): la production lamellaire dans la séquence de La Salle de saint- Martin In: le Brun-Ricalens F, editor. Productions lamellaires attribuées à l’Aurignacien. Luxembourg: MNHA; 2005 p. 375–92.

[135] Normand C, de Beaune SA, Costamagno S, Diot MF, Henry-Gambier D, Goutas N, et al Nouvelles données sur la séquence aurignacienne de la grotte d’Isturitz (communes d’Isturitz et de Saint-Martind’Abreroue: Pyrénées-Atlantiques) In: Evin J, editor. Congrés du centenaire de la Société préhistorique française: Un siècle de construction du discours scientifiquecen Préhistoire, 26éme Congrès préhistorique de France Aux Conceptions d’Aujourd’hui. 3 Paris: Société préhistorique française; 2007 p. 277–93.

[136] Bordes JG. Chatelperronian/Aurignacian interstratification at Roc de Combe and Le Piage: lithic taphonomy and archaeological implications. J Hum Evol. 2002;42(3):A7–A8. PubMed PMID: WOS:000175111600016.

[137] Sitlivy V, Chabai V, Anghelinu M, Uthmeier T, Kels H, Nita L, et al Preliminary reassessment of the Aurignacian in Banat (South-western Romania). Quatern Int. 2014;351:193–212. doi: 10.1016/j.quaint.2012.07.024 PubMed PMID: WOS:000345521100016.

[138] Bataille G. Der Übergang vom Mittel- zum Jungpaläolithikum auf der Halbinsel Krim und in der Kostenki-Borshchevo-Region am Mittel-Don Adaptionsstrategien spät-mittelpaläolithischer und frühjungpaläolithischer Gruppen. Cologne: University of Cologne; 2013.

[139] Roussel M, Soressi M, Hublin JJ. The Chatelperronian conundrum: Blade and bladelet lithic technologies from Quincay, France. J Hum Evol. 2016;95:13–32. doi: 10.1016/j.jhevol.2016.02.003 . 27260172 10.1016/j.jhevol.2016.02.003

[140] Barshay-Szmidt C, Eizenberg L, Deschamps M. Radiocarbon (AMS) dating the Classic Aurignacian, Proto-Aurignacian and Vasconian Mousterian at Gatzarria Cave (Pyrénées-Atlantiques, France). Paléo. 2013;23:1–42.

[141] Rios Garaizar J. Industria lítica y sociedad del paleolítico medio al superior en torno al Golfo de Bizkaia. Santander: PUbliCan, Ediciones de la Universidad de Cantabria; 2012. 561 p.

[142] Chiotti L. Les industries lithiques aurignaciennes de l’abri Pataud, Dordogne, France. Oxford: Archaeopress, BAR International Series; 2005. 349 p.

[143] Le Brun-Ricalens F. Réflexions préliminaires sur le comportement litho-technologique et l'occupation du territoire du pays des Serres à l'Aurignacien. Paléo. 1993;5(1):127–53. doi: 10.3406/pal.1993.1108

[144] Maillo-Fernandez JM. La Transición Paleolítico Medio-Superior en Cantabria: análisis tecnológico de la industria lítica de Cueva Morín. Madrid: UNED University; 2003.

[145] Teyssandier N, Bon F, Bordes JG. WITHIN PROJECTILE RANGE Some Thoughts on the Appearance of the Aurignacian in Europe. J Anthropol Res. 2010;66(2):209–29. PubMed PMID: WOS:000279139600003.

[146] Bataille G. Flakes and blades. The role of flake production in the Aurignacian of Siuren 1 (Crimea, Ukraine) In: Pastoors A, Peresani M, editors. Flakes Not Blades: The Role of Flake Production at the Onset of the Upper Palaeolithic in Europe. 5 Mattmann: Wissenschaftliche Schriften des Neanderthal Museums; 2012 p. 261–93.

[147] Maillo-Fernandez JM. Missing lithics: the role of flakes in the Early Upper Palaeolithic of the Cantabrian region (Spain) In: Pastoors A, Peresani M, editors. Flakes Not Blades: The Role of Flake Production at the Onset of the Upper Palaeolithic in Europe. 5 Mettmann: Wissenschaftliche Schriften des Neanderthal Museums; 2012 p. 69–84.

[148] Bolus M. Flake production in the Aurignacian of southwestern Germany: Some examples from the Swabian Jura In: Pastoors A, Peresani M, editors. Flakes Not Blades: The Role of Flake Production at the Onset of the Upper Palaeolithic in Europe. 5 Mettmann: Wissenschaftliche Schriften des Neanderthal Museums; 2012 p. 153–64.

[149] Normand C. L’Aurignacien de la salle de Saint-Martin (Grotte d’Isturitz, commune de Saint-Martin d’Arberoue, Pyrénéesatlantiques): donnés préliminaires sur l’industrie lithique recueillie lors des campagnes 2000–2002 In: Bon F, Maillo Fernandez JM, Ortega-Cobos D, editors. Autour des concepts de Protoaurignacien, d’Aurignacien archaïque, initial et ancien Unité et variabilité des comportements techniques des premiers groupes d’hommes modernes dans le sud de la France et le nord de l’Espagne. Madrid, Spain: UNED; 2006 p. 145–74.

[150] Hahn J. Aurignacien. Das ältere Jungpaläolithikum in Mittel- und Osteuropa. Köln: Böhlau (Fundamenta, 9); 1977.

[151] Conard N, Bolus M. The Swabian Aurignacian and its place in European Prehistory In: Bar-Josef O, Zilhão J, editors. Towards a Definition of the Aurignacian. Lisbon: IPA; 2006 p. 219–39.

[152] Tejero JM, Grimaldi S. Assessing bone and antler exploitation at Riparo Mochi (Balzi Rossi, Italy): implications for the characterization of the Aurignacian in South-western Europe. J Archaeol Sci. 2015;61:59–77. doi: 10.1016/j.jas.2015.05.003 PubMed PMID: WOS:000359877200006.

[153] Tsanova T. The beginning of the Upper Paleolithic in the Iranian Zagros. A taphonomic approach and techno-economic comparison of Early Baradostian assemblages from Warwasi and Yafteh (Iran). J Hum Evol. 2013;65(1):39–64. doi: 10.1016/j.jhevol.2013.04.005 . 23742933 10.1016/j.jhevol.2013.04.005

[154] Otte M, Kozłowski JK. La place du Baradostien dans l’origine du Paléolithique supérieur d’Eurasie. l'Anthropologie 2004;108:395–406.

[155] Becerra-Valdivia L, Douka K, Comeskey D, Bazgir B, Conard NJ, Marean CW, et al Chronometric investigations of the Middle to Upper Paleolithic transition in the Zagros Mountains using AMS radiocarbon dating and Bayesian age modelling. J Hum Evol. 2017;109:57–69. http://dx.doi.org/10.1016/j.jhevol.2017.05.011. 28688460 10.1016/j.jhevol.2017.05.011

[156] Conard N, Ghasidian E. The Rostamian cultural group and the taxonomy of the Iranian Upper Paleolithic In: Conard N, Drechsler P, Morales A, editors. Between Sand and Sea: the Archaeology and Human Ecology of Southwestern Asia. Tübingen: Kerns Verlag; 2011 p. 33–52.

[157] Ghasidian E, Bretzke K, Conard NJ. Excavations at Ghār-e Boof in the Fars Province of Iran and its bearing on models for the evolution of the Upper Palaeolithic in the Zagros Mountains. J Anthropol Archaeol. 2017;47:33–49. http://dx.doi.org/10.1016/j.jaa.2017.03.001.

[158] Goring-Morris N, Belfer-Cohen A. More than meets the eye Studies on upper Palaeolithic diversity in the near east. Oxford: Oxbow Books; 2003.

