# Supplementary material for: A critical assessment of the Protoaurignacian lithic technology at Fumane Cave and its implications for the definition of the earliest Aurignacian
Source: PLoS One. 2017 Dec 7;12(12):e0189241. doi: 10.1371/journal.pone.0189241 (PMC5720803; doi:10.1371/journal.pone.0189241)

S1 Fig. Plan view of the cave. Squares colored yellow are square meters where all cores, all tools and tool fragments, all complete and almost complete blades and bladelets, and all by-products deemed to have had a significant role in the reduction process were studied. Additionally, in squares colored brown all blades and bladelets greater than 1.5 cm regardless of the fragmentation index and all flakes with preserved butts greater than 2.0 cm were analyzed.

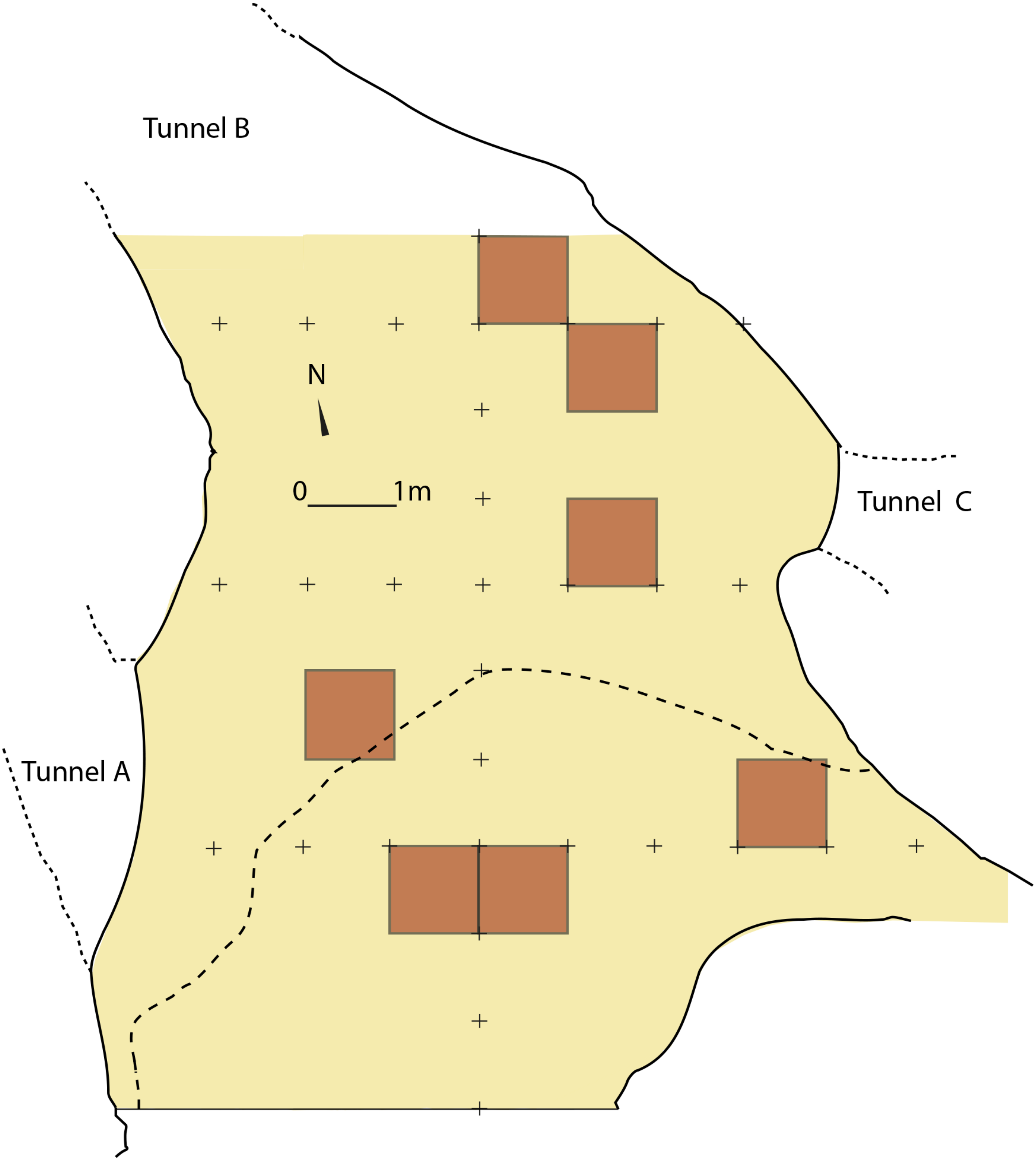

Supplement: S1 Fig — Squares colored yellow are square meters where all cores, all tools and tool fragments, all complete and almost complete blades and bladelets, and all by-products deemed to have had a significant role in the reduction process were studied. Additionally, in squares colored brown all blades and bladelets greater than 1.5 cm regardless of the fragmentation index and all flakes with preserved butts greater than 2.0 cm were analyzed. (PDF) [file pone.0189241.s002.pdf]
