# Supplementary material for: A critical assessment of the Protoaurignacian lithic technology at Fumane Cave and its implications for the definition of the earliest Aurignacian
Source: PLoS One. 2017 Dec 7;12(12):e0189241. doi: 10.1371/journal.pone.0189241 (PMC5720803; doi:10.1371/journal.pone.0189241)
Supplement: S2 Fig — Schematic drawings of semi-circumferential blade (a) and bladelet (b, e) cores, wide-faced flat blade-bladelet (c) and blade (h) cores, narrow-sided bladelet cores (d, i), transverse carinated bladelet core (f), and multi-platform bladelet core (g). See individual captions for interpretation of core reduction procedures and the legend for explanation of the symbols and graphic criteria used to draw cores (drawings: A. Falcucci). (PDF) [file pone.0189241.s003.pdf]

S2 Fig. Core diacritic analyses. Schematic drawings of semi-circumferential blade (a) and bladelet (b, e) cores, wide-faced flat blade-bladelet (c) and blade (h) cores, narrow-sided bladelet cores (d, i), transverse carinated bladelet core (f), and multi-platform bladelet core (g). See individual captions for interpretation of core reduction procedures, and the legend for explanation of the symbols and graphic criteria used to draw cores (drawings: A. Falcucci).

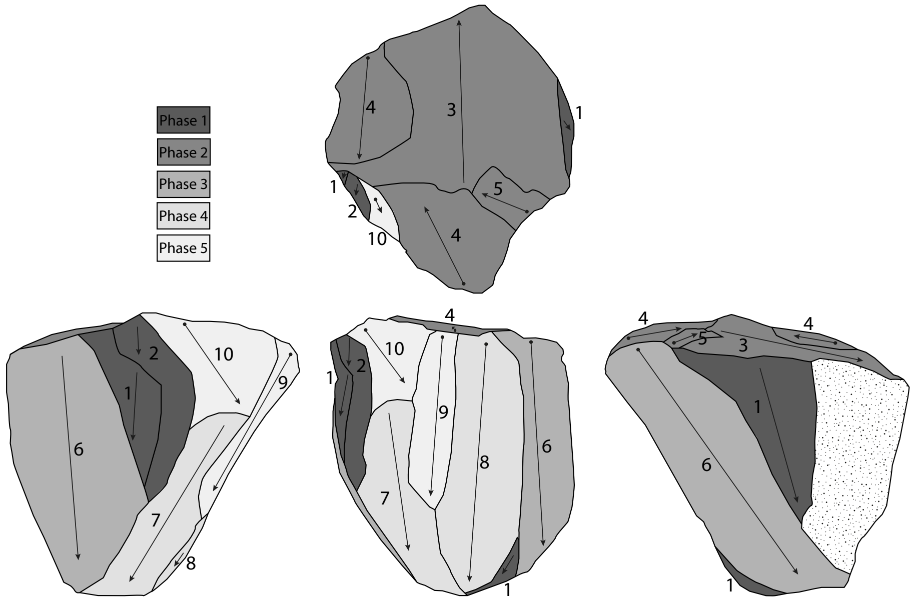

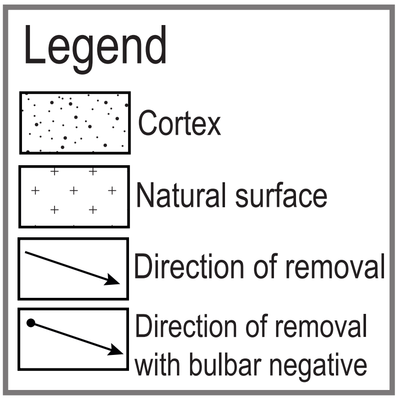

a. Semi-circumferential blade core. Phase 1: core decortication; Phase 2: shaping of the striking platform; Phase 3: removal of a naturally backed blade; Phase 4: blade production; Phase 5: hinged removal (9) and failed attempt to strike a technical transverse flake (10).

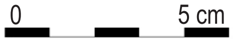

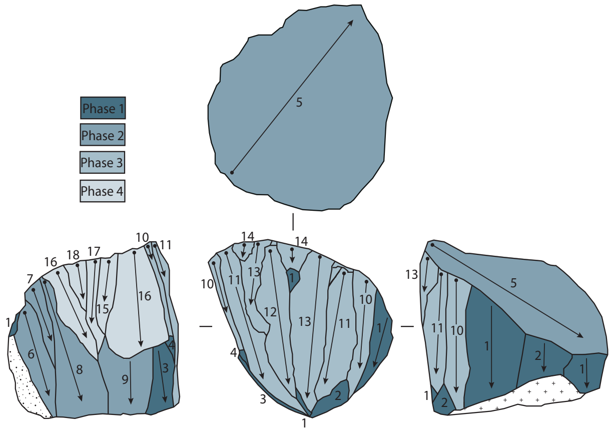

b. Semi-circumferential bladelet core. Phase 1: early stage of core preparation and isolation of a narrow surface (see removal 3). The related bladelet production is no longer visible; Phase 2: removal of a total core tablet and new isolation of the flaking surface; Phase 3: bladelet production; Phase 4: last attempt to reshape and isolate a narrow surface.

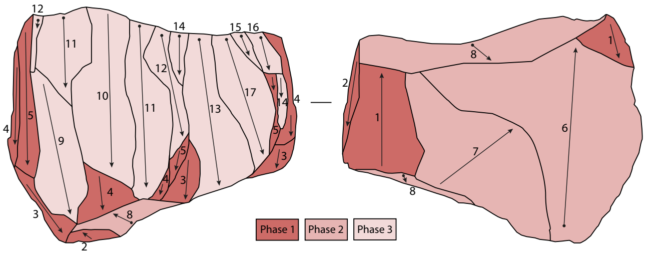

c. Wide-faced flat blade-bladelet core. Phase 1: early phase of blank production; Phase 2: maintenance operations at the core back and base and removal of a core tablet; Phase 3: simultaneous blade-bladelet production.

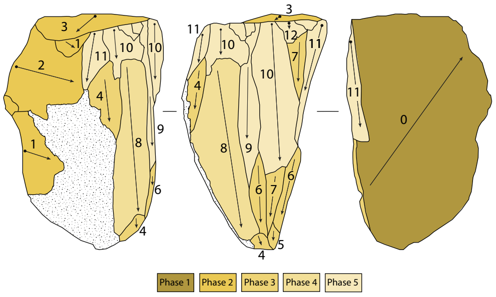

d. Narrow-sided bladelet core. Phase 1: core blank is a thick cortical flake; Phase 2: creation of a dorsal crest and of a striking platform; Phase 3: bladelet production; Phase 4: a lateral blade is detached; Phase 5: last set of hinged bladelets.

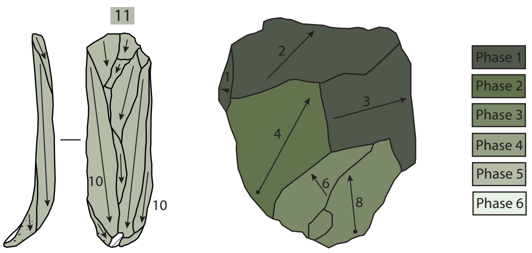

e. Semi-circumferential bladelet core. Phase 1: core decortication; Phase 2: re-preparation of the striking platform and maintenance of the core flank; Phase 3: Isolation of a narrow and convex surface; Phase 4: bladelet production; Phase 5: new isolation of an adjacent surface (see also the related plunging blade 11); Phase 6: bladelet production and last failed attempt to accentuate the transversal convexity (14).

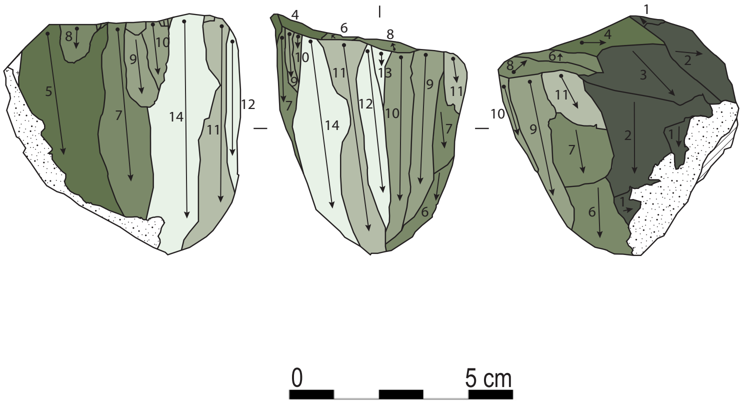

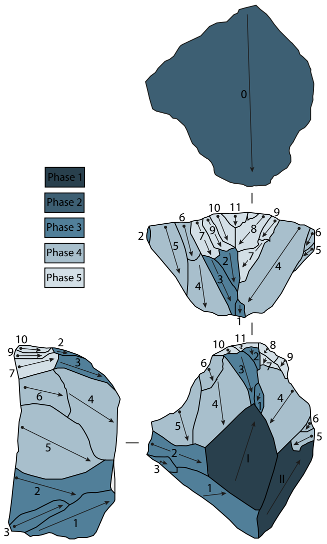

f. Transverse carinated bladelet core. Phases 1 & 2: core blank is a thick flake; Phase 3: lateral isolation of the flaking surface and early bladelet production; Phase 4: new isolation of the flaking surface that gives the core a nosed shape; Phase 5: last set of hinged removals.

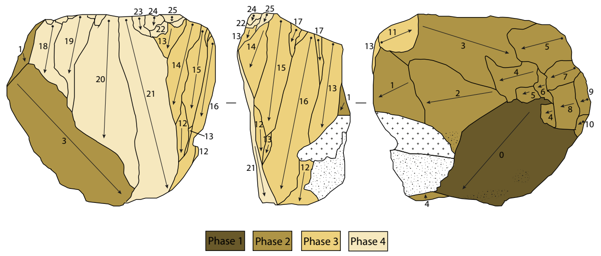

g. Multi-platform bladelet core. Phase 1: core blank is a flake; Phase 2: shaping of a dorsal crest and preparation of the core flanks; Phase 3: bladelet production based on the narrow face; Phase 4: re-orientation of the core and a set of broad bladelet removals on a broad, disjointed face.

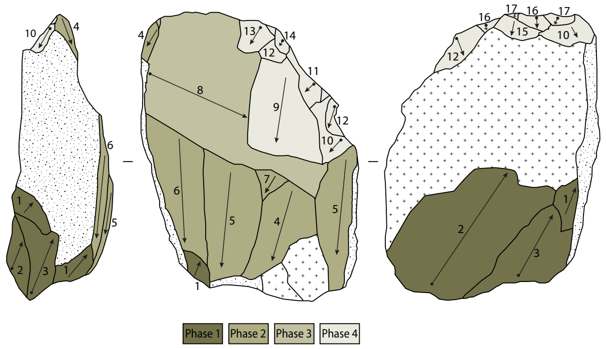

h. Wide-faced flat blade core. Phase 1: maintenance operations on the core base and back; Phase 2: blade production; Phase 3: strike of an orthogonal rejuvenation flake; Phase 4: re-preparation of the striking platform by short hinged flakes (faceted platform) and failed attempt to pursue the blank production.

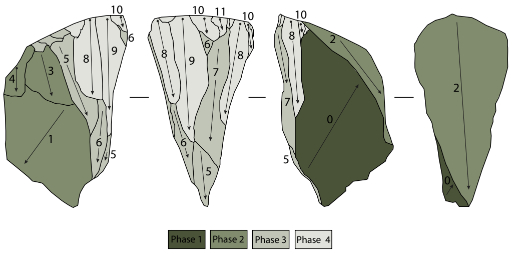

i. Narrow-sided bladelet core. Phase 1: core blank is a flake; Phase 2: shaping of the core flank and creation of the striking platform; Phase 3: bladelet production; Phase 4: last set of bladelet removals, some of them hinged.
