# Supplementary material for: A critical assessment of the Protoaurignacian lithic technology at Fumane Cave and its implications for the definition of the earliest Aurignacian
Source: PLoS One. 2017 Dec 7;12(12):e0189241. doi: 10.1371/journal.pone.0189241 (PMC5720803; doi:10.1371/journal.pone.0189241)

S3 Fig. Comparison between the distribution of blade thickness values (in millimeters; blue) and bladelet thickness values (in millimeters; green).

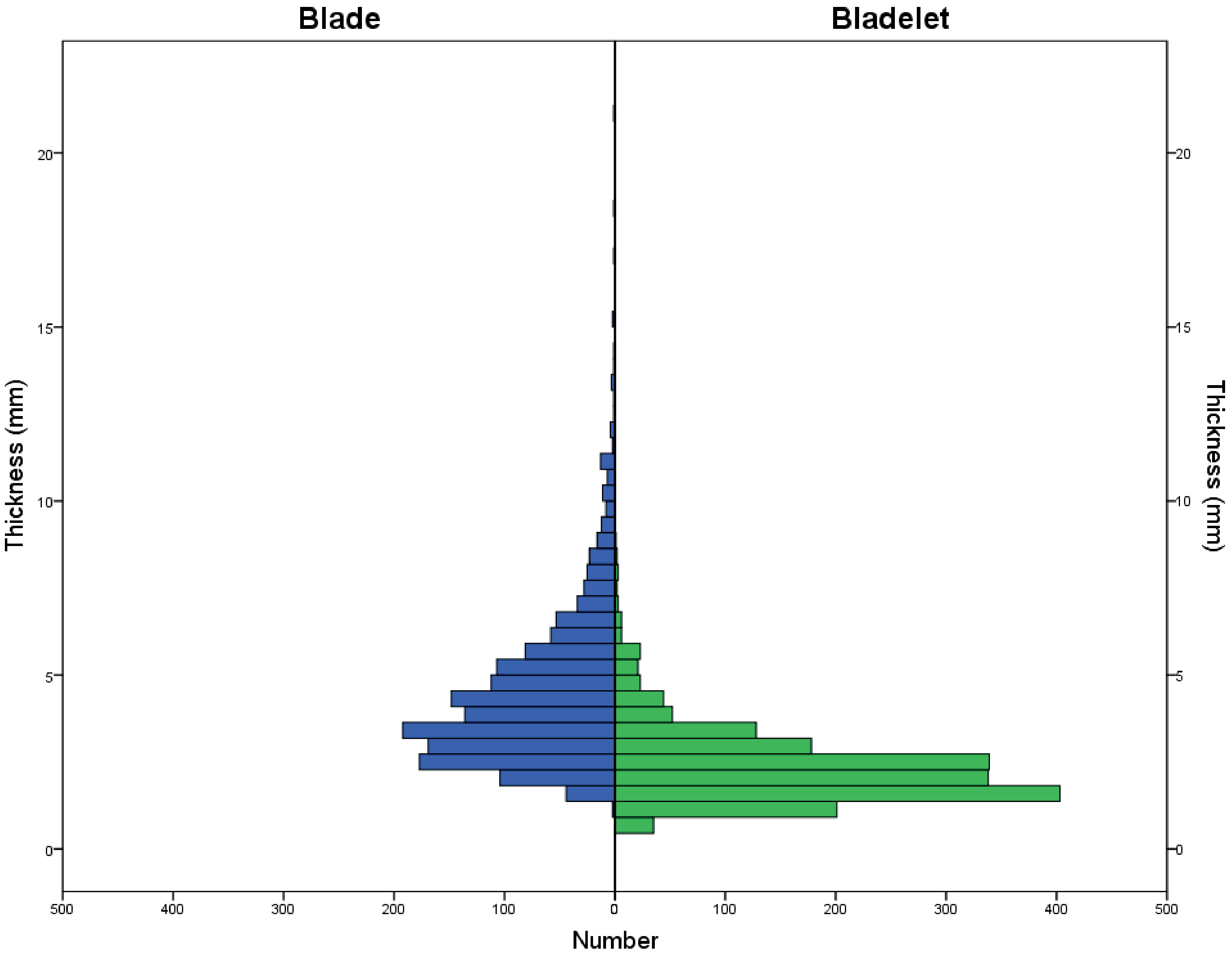

Supplement: S3 Fig — (PDF) [file pone.0189241.s004.pdf]
