# Supplementary material for: A critical assessment of the Protoaurignacian lithic technology at Fumane Cave and its implications for the definition of the earliest Aurignacian
Source: PLoS One. 2017 Dec 7;12(12):e0189241. doi: 10.1371/journal.pone.0189241 (PMC5720803; doi:10.1371/journal.pone.0189241)
Supplement: S1 Table — SE: standard error; SD: standard deviation. (PDF) [file pone.0189241.s005.pdf]

S1 Table. Summary of length measurements across complete blanks (flakes, blades, and bladelets together) with different grades of cortex coverage. SE: standard error; SD: standard deviation.

	Number	Range	Mean	SE	SD	25 prcntl	Median	75 prcntl
**1-33%**	299	11.9 to 103.3	44.5	0.89	15.43	33.7	42.8	51.6
**33-66%**	94	19.7 to 95.0	46.0	1.64	15.95	34.3	44.1	56.3
**66-99%**	51	17.8 to 91.0	47.0	2.45	17.49	32.5	43.4	57.2
**100%**	31	15.8 to 75.0	43.6	2.57	14.33	36.5	42.1	53.6
