# Supplementary material for: A critical assessment of the Protoaurignacian lithic technology at Fumane Cave and its implications for the definition of the earliest Aurignacian
Source: PLoS One. 2017 Dec 7;12(12):e0189241. doi: 10.1371/journal.pone.0189241 (PMC5720803; doi:10.1371/journal.pone.0189241)
Supplement: S2 Table — SE: standard error; SD: standard deviation. (PDF) [file pone.0189241.s006.pdf]

S2 Table. Summary of metric attributes of blades made from Oolithic flint and blades made from all other raw material types. SE: standard error; SD: standard deviation.

	Number	Range	Mean	SE	SD	25 prcntl	Median	75 prcntl
**Oolithic**								
**Length**	4	69.0 to 95.0	78.6	6.0	12.0	69.3	75.2	91.3
**Width**	41	12.6 to 31.5	22.1	0.83	5.38	17.0	21.8	26.4
**Thickness**	41	1.7 to 12.0	6.0	0.37	2.35	4.1	5.8	7.4
**Other**								
**Length**	417	24.2 to 102.5	49.4	0.64	13.06	39.8	47.5	57.8
**Width**	1543	12.1 to 35.8	16.4	0.10	3.90	13.6	15.3	18.0
**Thickness**	1543	1.1 to 21.0	4.4	0.06	2.21	2.9	3.9	5.4
