# Supplementary material for: A critical assessment of the Protoaurignacian lithic technology at Fumane Cave and its implications for the definition of the earliest Aurignacian
Source: PLoS One. 2017 Dec 7;12(12):e0189241. doi: 10.1371/journal.pone.0189241 (PMC5720803; doi:10.1371/journal.pone.0189241)
Supplement: S3 Table — Complete technical flakes with undetermined scars (n = 6) are excluded. SE: standard error; SD: standard deviation. (PDF) [file pone.0189241.s007.pdf]

S3 Table. Summary of length measurements across complete technical flakes with blade, bladelet, and simultaneous blade-bladelet scars. Complete technical flakes with undetermined scars (n=6) are excluded. SE: standard error; SD: standard deviation.

	Number	Range	Mean	SE	SD	25 prcntl	Median	75 prcntl
**Blade**	16	36.2 to 116.0	52.8	4.80	19.20	42.9	49.0	51.8
**Bladelet**	55	10.9 to 60.0	39.4	1.21	9.09	33.6	38.7	44.4
**Blade-bladelet**	10	36.0 to 76.0	50.0	4.31	13.66	38.5	46.8	59.9
